# Assessing the determinants of out-of-pocket health expenditures among Cambodian households in informal employment using survey data

**DOI:** 10.1186/s12939-025-02394-6

**Published:** 2025-01-31

**Authors:** Andrea Hannah Kaiser, Sovathiro Mao, Jesper Sundewall, Marlaina Ross, Sokunthea Koy, Searivoth Vorn, Pichenda Koeut, Bjoern Ekman

**Affiliations:** 1 Department of Clinical Sciences, Malmoe (IKVM), Division of Social Medicine and Global Health (SMGH), CRC, Lund University, Jan Waldenstroems Gata 35, Malmoe, Sweden; 2 General Secretariat for the National Social Protection Council, Ministry of Economy and Finance of Cambodia, Street 92, Phnom Penh, 120211 Cambodia; 3 HEARD, University of KwaZulu-Natal, Durban, South Africa; 4 Causal Design, FACTORY Phnom Penh, Phnom Penh, 1159 NR2 Cambodia; 5 Deutsche Gesellschaft für Internationale Zusammenarbeit (GIZ) GmbH Cambodia, Improving Social Protection and Health Project, Sayon Building, Samdach Pan Ave No. 41, Phnom Penh, 12211 Cambodia

**Keywords:** Financial protection, Out-of-pocket health expenditures, Out-of-pocket budget share, Universal health coverage, Population coverage, Informal employment, Low- and middle-income countries, Cambodia

## Abstract

**Background:**

As the deadline for the Sustainable Development Goals approaches, financial protection in Cambodia remains inadequate, especially for nonpoor informal workers lacking formal social health protection coverage or access to other prepayment schemes. This exposes them to high out-of-pocket health expenditures (OOPE) and related financial hardship. To better understand the drivers behind these expenditures, our study aims to model their *healthcare*, *health*, and *social* determinants and to assess their relative importance.

**Methods:**

In 2023, we conducted a cross-sectional multistage clustered sampling survey across seven Cambodian provinces, surveying 3,254 households engaged in informal employment and not covered by any formal social health protection scheme. The survey gathered information on households’ use of outpatient and inpatient care and associated OOPE. We employed generalized linear models (GLMs) to analyse the *healthcare*, *health*, and *social* determinants of OOPE and the OOPE budget share (the proportion of total annual household consumption expenditure spent on OOPE) and applied Shapley decomposition analysis to quantify the relative contributions of these determinants to the explained variance in our outcomes.

**Results:**

*Healthcare* variables were the dominant contributors to the explained variance in all outcomes (41.36–50.73%), followed by *health* factors. While several *social* variables were significant, only the wealth quintile made notable contributions to explaining variance in our outcomes. The key *healthcare* contributors included the sector type and level of care, and the number of outpatient medications. Important *health* contributors included illness severity and the presence of chronic illnesses or noncommunicable diseases.

**Conclusions:**

Our findings emphasize the necessity of integrating nonpoor informal workers and their dependents into formal prepayment schemes to reduce OOPE and enhance financial protection on Cambodia’s path toward universal health coverage. Strategically engaging with private providers and pharmacies to improve access to essential services and medicines, coupled with the implementation of an effective referral system are important policy considerations to this end. Further research is needed on how *health* determinants are modifiable with policy interventions. Our findings can assist the Cambodian government in advancing its universal health coverage goals and offer insights for other countries aiming to extend coverage to similar population groups.

**Supplementary Information:**

The online version contains supplementary material available at 10.1186/s12939-025-02394-6.

## Background

As we approach the deadline for the Sustainable Development Goals, universal health coverage (UHC) remains a central global policy priority. Despite global improvements in service coverage, the 2023 UHC monitoring report highlighted persistent and growing challenges related to financial protection, particularly the burden of financial hardship due to high out-of-pocket health expenditures (OOPE) [1]. These challenges are pronounced in low- and middle-income countries (LMICs), where extensive informal employment hinders the collection of adequate public healthcare funding from direct taxes [2–4]. To date, few LMICs have effectively extended formal prepayment schemes to nonpoor workers in informal employment (hereinafter referred to as informal workers) and their dependents, often leaving this key demographic without coverage, and thus vulnerable to financial hardship and the necessity of employing adverse coping strategies [2, 3, 5]. This situation represents a significant equity challenge. A major criticism of the Millenium Development Goals was their insufficient focus on equity [6]. Current evidence similarly indicates that UHC initiatives that do not explicitly prioritize equity may lead to persistent or even widening disparities [7–9] – despite the inclusion of utilization relative to need and equity in finance as UHC coverage goals [10].

With 93.1% of Cambodia’s economically active population engaged in informal employment [5], the country lags in extending formal prepayment scheme coverage to its nonpoor informal workers and their dependents, resulting in lower population coverage and financial protection outcomes compared to its Southeast Asian peers [11]. In 2021, OOPE accounted for approximately 55% of Cambodia’s current health expenditure, one of the highest proportions globally [12]. Furthermore, despite extensive reform efforts and expansions of coverage through the Health Equity Fund (HEF) to poor and near-poor households and the National Social Security Fund (NSSF) to formal workers, civil servants, and their dependents, approximately 59% of the population—predominantly nonpoor informal workers and their dependents—remains uncovered [13]. This group, referred to as uncovered households throughout this manuscript, often faces precarious work and economic conditions, exacerbating their vulnerability to health-related financial shocks [5]. Previous research has shown that uncovered households experience elevated levels of catastrophic health expenditure [14, 15].

To address gaps in global coverage and better integrate nonpoor informal workers and their dependents into prepayment schemes, recent research has suggested desirable reform directions including, for example, increased reliance on public revenues [2–4, 11]. Moreover, numerous studies have analysed the determinants of OOPE and financial protection in LMICs [16–34]. For example, studies conducted across different Asian countries identified a range of *social* variables such as age, education, household size, dependencies, occupation, wealth quintile, and geographical domain as determinants of these outcomes [16, 19–24, 27–29, 32–38]. Although many of these studies also included *health* characteristics such as chronic illnesses and disabilities [19–24, 36, 38, 39], only few considered *healthcare* characteristics, despite findings consistently highlighting their significance [16, 24, 28, 34, 36]. Additionally, there is a notable scarcity of research specifically focusing on nonpoor informal workers and the determinants of the OOPE budget share, a continuous measure relating OOPE to a household’s overall expenditure [40]. Most existing studies also restrict their analysis to identifying significant associations without assessing the relative importance of these determinants.

Against this background, our study has two primary aims: (1) to model the *healthcare*, *health*, and *social* determinants of OOPE and the OOPE budget share among uncovered households in Cambodia, and (2) to assess the relative importance of these determinants. This research directly supports ongoing efforts under Cambodia’s UHC Roadmap 2024–2035 [41]. By addressing several gaps in the current literature, our findings also strengthen the global evidence base regarding the determinants of OOPE and the OOPE budget share. Furthermore, our findings offer insights for other LMICs with contexts of high informal employment, where policymakers face similar challenges in designing and implementing policies and interventions to enhance informal workers’ financial protection.

## Methods

### Data

We conducted a cross-sectional survey of 3,254 households, encompassing 15,421 individuals, in June and July 2023 in seven Cambodian provinces. Our study population included uncovered Cambodian households who sought outpatient, inpatient, or preventive services, with households selected using a multistage clustered sampling design. Additional details are provided in Additional File 1. The respondents were heads of household who provided information on household demographics, assets, and consumption; household member health and disability status and their utilization of preventive, outpatient, and inpatient services and associated OOPE based on a structured survey questionnaire. Questions on the utilization of outpatient and preventive services were based on a 30-day recall period, while questions on inpatient care followed a 12-month recall period. These recall periods align with Cambodia’s Socio-Economic Survey methodology, facilitating comparability with national data, and reflect established survey practices that balance accurate expenditure reporting with the relative rarity of hospitalizations [40]. We collected data on 5,234 outpatient, 494 preventive, and 714 inpatient visits. The demographic and socioeconomic survey questions were aligned with those of the Cambodia Socio-Economic Survey, and the questions on healthcare-seeking were aligned with those of a previous study [42]. Following pilot testing, only minor refinements to question wording were made as most survey items had been previously validated. Illness categories were adapted from the Institute of Health Metrics and Evaluation (IHME) Burden of Disease study, whereby we included detailed response options for each of the categories outlined by the IHME: communicable diseases; noncommunicable diseases (NCDs); maternal, neonatal, and nutritional diseases (MNNDs); and injuries, violence, self-harm, and accidents (injuries) [43]. Data collection was conducted by a local firm over eight weeks. The data collection process adhered to standardized protocols to ensure high data quality, including comprehensive training and pilot testing, and continuous supervision with repeat interviews and regular data quality checks.

### Variables

We modelled total OOPE as a continuous outcome measure, defined as household annual OOPE in monetary terms at the point of service delivery for outpatient and inpatient care, excluding any third-party payments. We also created separate models for outpatient and inpatient OOPE to account for their distinct cost structures. Additionally, we modelled the OOPE budget share, or OOPE as a fraction of total annual household consumption expenditure [44]. This continuous financial protection indicator provides an alternative to catastrophic health expenditure by not relying on specific thresholds to measure financial hardship, avoiding the controversies related to the arbitrary selection of such thresholds [45, 46]. Table 1 in Additional file 1 details the measurement of all outcome variables.

**Table 1:** Sample characteristics at the household level

	Value	Standard error
**Demographic characteristics**		
Household size	3.95	0.08
Share head of household who is female	35.56%	4.06
Share household members under 5	9.93%	0.62
Share household members over 60	11.36%	0.79
Share household members with primary education or lower	57.40%	1.40
Employment ratio	46.46%	0.81
**Health characteristics**		
Share household members with chronic illness	18.30%	0.95
Share household members with disability	9.83%	0.68
Share household members in self-reported health < good	52.32%	1.52
**Socioeconomic characteristics**		
Mean (median) total household consumption expenditure	$5,926.83 ($4,926.83)	116.57
**OOPE and financial protection indicators**		
Share with any OOPE	99.26%	0.20
Mean (median) total OOPE	$475.30 ($148.37)	31.70
Mean (median) OOPE for outpatient care	$372.76 ($118.70)	26.45
Mean (median) OOPE for inpatient care	$517.18 ($128.05)	54.95
Mean (median) OOPE budget share	7.84% (3.11%)	0.49
Incidence of catastrophic health expenditure (10%)	24.24%	1.53
Incidence of catastrophic health expenditure (25%)	5.98%	1.04
Incidence of impoverishment (national poverty line)	6.67%	0.75
Share borrowing or selling land/assets for healthcare	5.71%	0.86
Reduced food expenditure for outpatient care (inpatient)	28.28% (42.60%)	1.85 (5.33)
Reduced other essential spending for outpatient (inpatient)	18.64% (30.31%)	1.28 (2.66)
Reduced education spending for outpatient (inpatient)	2.48% (5.32%)	1.07 (3.97)
**Care-seeking characteristics**		
Share who sought outpatient care	96.76%	0.61
Share who sought inpatient care	22.16%	1.03
Share who sought both outpatient & inpatient care	18.92%	0.99
Share obtaining any outpatient medications	94.43%	1.21
Mean (median) number of outpatient medications (30 days)	5.03 (4)	0.14
Share who sought care in public sector	18.85%	1.09
Share who sought care in private sector	92.10%	1.11
Share who sought care in nonmedical sector	2.11%	0.56
Share who sought care overseas	0.37%	0.14
Share with maternal, neonatal, and nutritional diseases	8.98%	0.73
Share with communicable diseases	67.46%	1.90
Share with noncommunicable diseases	47.72%	1.53
Share with injuries	4.50%	0.41
Mean days lost to illness/injury	8.65	0.76

Abbreviations: OOPE = out-of-pocket health expenditures

**Fig. 1:**
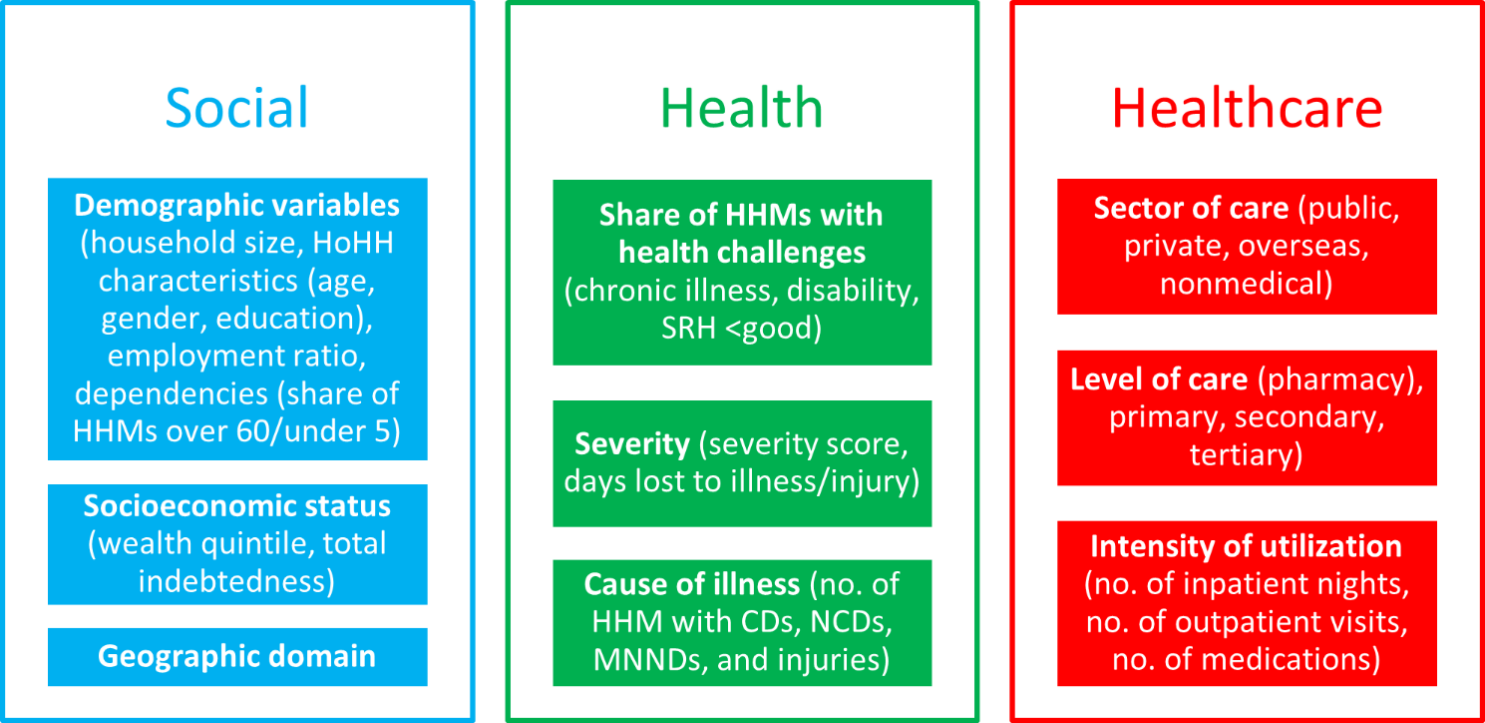
*Healthcare*, *health*, and *social* explanatory variables in regression and decomposition analysis. Adapted from [24]. Abbreviations: CD = communicable disease; HHM = household member; HoHH = head of household; MNNDs = maternal, neonatal, nutritional diseases; NCDs = noncommunicable diseases; SRH = self-rated health.

We employed a systematic approach to select explanatory variables. First, we developed an initial list based on the Cambodian context and available data. This list was then refined through a comprehensive literature review of over 100 studies on the determinants of OOPE and financial protection in LMICs, with key studies and their findings detailed in Additional file 2. We further validated our selection through established theoretical frameworks, notably Grossman’s demand for health model and Aday and Andersen’s model of healthcare utilization [47–50]. The final selection of explanatory variables was confirmed through empirical testing of model specifications. Variables were then categorized into three groups: (i) *healthcare* variables; (ii) *health* variables; and (iii) *social* variables. This categorization reflects the varying degrees of influence public policy can exert, as suggested by previous research [24]. Figure 1 illustrates the final selection and grouping of variables and Table 2 in Additional file 1 provides further details on all explanatory variables, including their measurements and expected relationships with our outcomes.

### Data analysis

#### Data management

The unit of analysis was the household, consistent with internationally standardized methods for measuring financial protection in health [40, 51, 52]. We aggregated healthcare utilization and expenditure data, collected at the visit-level, to the household level, and annualized data points with recall periods of less than 12 months using time-neutral annualization factors. We excluded households that sought exclusively preventive care (40 observations). This decision was based on the rationale that preventive care typically follows different utilization and spending patterns from curative care. Preventive care is typically planned and discretionary, with different price elasticity patterns compared to curative care sought for acute or chronic conditions. Including preventive care could therefore confound our analysis of the *healthcare*, *health*, and *social* determinants of OOPE and the OOPE budget share. The grouping of healthcare providers into sectors and levels detailed in Tables 3a and 3b in Additional file 1 followed the Cambodian Ministry of Health’s framework [53], and the grouping of illness categories from our survey was aligned with the IHME approach (Additional file 1, Table 4) [43]; however, we maintained MNNDs and communicable diseases as distinct categories. We converted all monetary values into 2023 US dollars ($) using the average 2023 exchange rate of 4,100 Khmer Riel to $1.

### Statistical analysis

We utilized descriptive statistics to characterize the study sample, including demographic characteristics, healthcare utilization, associated OOPE, and the level of financial protection.

All outcome measures exhibited severe right-skewness. For instance, the top 1% of spending households accounted for 21% of total OOPE, and the top 10% accounted for 57% (Additional file 1, Table 5). We applied a systematic approach to determine the most suitable model for each outcome, comparing ordinary least squares (OLS) on the natural log of outcomes with generalized linear models (GLMs) with log links [54] based on kurtosis checks of the residuals, Akaike and Bayesian information criteria (AIC and BIC), predicted outcome means, and scatterplots. GLMs slightly outperformed both the heteroskedastic and homoskedastic log OLS models in our data. However, our conclusions held across both log OLS models and GLMs, indicating the robustness of our findings.

Empirical tests confirmed that the log link and gamma family were the best fit for our data [54, 55]. For the link function, we used a Box-Cox approach to identify the most symmetric distribution for the dependent variables, confirming the log link as the preferred choice (λ close to 0 for all outcomes). We used modified Park tests to empirically test the relationship between the mean and the variance of the error term in the GLMs; for all outcomes, the estimated coefficient was close to the gamma family’s integer value of 2 (ranging from 1.83 to 1.98). AIC and BIC further validated the log link with the gamma family as the best fit, showing the lowest values among the tested links and families.

We employed Shapley decomposition to quantify the relative importance of the explanatory variables. Shapley decomposition distributes a regression model’s goodness-of-fit measure (R^2^) across explanatory variables, expressing contributions as ratios to the overall explained variance [56]. Marginal contributions are calculated by sequentially eliminating variables and then averaging these effects across all possible elimination sequences [57]. Shapley decomposition is additive and path-independent, ensuring that the sum of the individual variable contributions equals the total explained variance and that the order of variable entry does not influence the assigned contributions. Additionally, it satisfies both monotonicity and equal treatment properties, and considers the correlation among variables [56, 57]. Israeli (2007) provided a detailed description of the procedures for determining the exact contributions of explanatory variables to the R^2^ of a linear regression model [58]. While GLMs provided a slightly better fit for our skewed data, we employed OLS for the Shapley decomposition due to its established theoretical foundation and validation for variance decomposition of linear models and the clear interpretation of R² as proportion of the explained variance [58]. The use of log-transformed variables helped linearize relationships and reduce the influence of extreme values, making OLS more appropriate. Additionally, as noted above, our conclusions held between the GLM and OLS models, suggesting that our decomposition results are not substantially biased by the choice of OLS.

Contributions to the R^2^ are calculated for our three defined groups of explanatory variables – *healthcare*, *health*, and *social* – as well as for the individual variables within these groups. We employed a stepwise approach to decomposition: starting with *social* characteristics alone (Specification 1), then adding *health* characteristics (Specification 2), and finally incorporating *healthcare* characteristics (Specification 3). This approach illustrates how the inclusion of additional variables shifts the allocated contributions among variables and groups. The full econometric model, utilizing OLS regression, is structured as follows [24]:

1 $$\begin{aligned}Y&={\beta\:}_{0}+\:\beta\:*healthcare\:variables\\&\quad+\:\gamma\:*health\:variables\\&\quad+\:\delta\:*social\:variables+\:\epsilon\end{aligned}$$

Statistical analyses were conducted using Stata 18.0. We considered a p-value of less than 0.05 to indicate statistical significance. We transformed all outcomes and two explanatory variables (severity score and days lost to illness/injury) using a natural logarithm to manage skewness in the Shapley decomposition. Data for the models estimating OOPE for outpatient and inpatient care were restricted to only households where at least one member sought the respective type of care.

We applied sampling weights to the summary statistics and GLM regressions and addressed within-cluster correlation with clustered standard errors in Shapley decomposition. Additionally, we employed bootstrapping with 1,000 replications to estimate confidence intervals in the decomposition, allowing us to compare individual and group contributions. Since R^2^ generally increases with each additional explanatory variable, it is not possible to estimate the significance of the individual covariate contributions [58]. To assess the influence of extreme values, we ran models with both winsorized and non-winsorized versions (top 1% and top 5%) of the outcome variables. The model results were robust across these comparisons, leading us to select the non-winsorized variables for our final analyses. Finally, we performed a comparative validation with the data from the 2023 and 2021 Cambodia Socio-Economic Surveys (Additional file 1, Table 6). This comprehensive validation strategy, combining bootstrapped confidence intervals, sensitivity analyses for extreme values, and external data comparison, strengthens the statistical validity of our results.

## Results

### Characteristics of the study sample

Table 1 outlines the characteristics of the surveyed households. The average household size was approximately 4 members, with 9.95% under 5 years old and 11.36% over 60. Educational attainment was low, and a considerable fraction of households reported health challenges, with 18.3% of all individuals surveyed suffering from a chronic illness and 9.83% living with disabilities. Approximately 96.76% of households sought outpatient care, 22.16% sought inpatient care, and 18.92% sought both. Households predominantly sought care at private providers (92.10%). Medication usage was also high, with 94.43% of households obtaining medications. Among these, the average household consumed 5.03 medications in the previous 30 days. Communicable diseases were the most prevalent illness, affecting 67.46% of households, followed by NCDs at 47.72%, MNNDs at 8.98%, and injuries at 4.50%. Nearly all households (99.26%) incurred OOPE, with annual expenditures averaging $475.30. At $372.8, average annual OOPE for outpatient care were lower than for inpatient care ($517.2). When including OOPE in total household consumption expenditure calculations (standard approach), the OOPE budget share was 7.84%, and catastrophic health expenditure affected 24.24% of households at the 10% threshold and 5.98% of households at the 25% threshold. In sensitivity analyses excluding OOPE from total household consumption expenditure, these estimates increased to 12.03% for the OOPE budget share, with 25.72% of households experiencing catastrophic expenditure at the 10% threshold and 9.22% at the 25% threshold. These higher estimates suggest that the standard practice of including OOPE in total household consumption expenditure calculations may provide conservative estimates of the financial burden of healthcare on households.

Additionally, 6.67% of households fell below the national poverty line due to OOPE, and 5.71% were forced to borrow or sell assets to afford healthcare. Coping strategies were common and included reducing food expenditure (28.28% for outpatient and 42.60% for inpatient care) and other essential spending (18.64% for outpatient, 30.31% for inpatient care).

### Determinants of OOPE and the OOPE budget share

Table 2 presents the GLM coefficients and marginal effects for total OOPE and the OOPE budget share, with the results for OOPE for outpatient and inpatient care available in Table 3. Starting with *healthcare* variables, the sector of care strongly influenced total OOPE. Compared with those seeking public outpatient care, households utilizing private outpatient, private inpatient, or overseas care experienced significant increases in OOPE ($310.95, $340.46, and $672.49, respectively; all *p* < 0.001). In comparison with pharmacies, access to higher levels of care, such as primary, secondary, or tertiary care also significantly elevated total OOPE, with tertiary care showing the greatest increase at $702.21 (*p* < 0.001). Among the variables indicating healthcare utilization intensity, only the number of medications obtained had a significant effect on total OOPE, with each additional medication increasing OOPE by $2.20 (*p* < 0.001). Concerning the *health variables*, the presence of chronic illness and increases in both perceived illness severity and the number of days lost to illness/injury within a household significantly elevated total OOPE. For example, chronic illnesses were associated with higher total OOPE of $193.26 (*p* < 0.05) and increased severity of $81.52 (*p* < 0.001). In contrast, the presence of members with a disability and in self-rated health below good did not significantly affect OOPE. Regarding illness type, households with members suffering from NCDs or injuries experienced increases in OOPE of $72.95 and $144.52, respectively (both *p* < 0.001) compared to households with members experiencing communicable diseases. Finally, among the *social* variables, the age and education level of the head of household (HoHH) significantly affected total OOPE. Specifically, every additional year of age was associated with an increase of $3.44 in total OOPE (*p* < 0.05) and households headed by someone with at least primary education incurred an average of $93.19 more than those without any formal education (*p* < 0.001). A clear socioeconomic gradient was evident, with OOPE increasing significantly in higher wealth quintiles. For example, the middle and richest quintiles experienced increases of $207.22 and $485.53, respectively, compared to the lowest quintile (*p* < 0.001). Geographic location also significantly affected total OOPE, with households in other urban and rural areas spending $318.0 and $194.70 more, respectively, than those in the capital (*p* < 0.001). Conversely, a higher employment ratio within a household was associated with a significant reduction in total OOPE of $120.44 (*p* < 0.05).

The patterns observed for outpatient and inpatient OOPE, as well as the OOPE budget share, generally aligned with those of total OOPE, albeit with several differences in significance and direction of effects. Across *healthcare* variables, private sector use, higher levels of care, and the number of medications consistently demonstrated significant positive relationships with outpatient and inpatient OOPE and the OOPE budget share, as for total OOPE. A notable difference was the number of inpatient nights: each additional night significantly increased inpatient OOPE by $82.49 (*p* < 0.001), while this relationship was not observed in other models. The analysis of *health* variables showed that chronic illness and NCDs significantly increased the OOPE budget share and OOPE for outpatient care but not for inpatient care. Interestingly, an increase in the number of household members with MNNDs significantly decreased outpatient OOPE by $237.60 (*p* < 0.001) while increasing it for inpatient care by $338.40 (*p* < 0.001). Notably, perceived severity did not influence inpatient OOPE significantly, in contrast to its significant effect in all other models. Injuries were significantly associated with all outcomes (*p* < 0.001) except outpatient OOPE. Finally, in terms of *social* variables, unlike the results for total OOPE, a larger household size significantly reduced the budget share by 0.47% and OOPE for inpatient care by $38.88 (both *p* < 0.05) per additional member. The employment ratio was associated with significant reductions in the OOPE budget share (*p* < 0.05) but not with inpatient or outpatient OOPE. The socioeconomic gradient, which we consistently observed across most outcomes, was reversed for the budget share, with higher wealth quintiles spending less on OOPE as a proportion of total household consumption expenditure than the poorest quintile (*p* < 0.05 to < 0.001).

**Table 2:** GLM estimates of total OOPE and the OOPE budget share

	Total OOPE						OOPE budget share							
	Coef	SE	*p*-value	Marginal effect	SE	*p*-value	Coef	SE	*p*-value	Marginal effect	SE		*p*-value	
**Healthcare determinants**														
*Sector of care (Ref: Public outpatient)*														
Public inpatient	0.27	0.20	0.18	$67.39	52.35	0.18	0.16	0.20	0.43	0.64%	0.01		0.45	
Private outpatient	0.88	0.12	0.00	$310.95	33.39	0.00	0.84	0.12	0.00	4.98%	0.01		0.00	
Private inpatient	0.93	0.20	0.00	$340.46	81.86	0.00	0.90	0.19	0.00	5.53%	0.01		0.00	
Overseas	1.40	0.41	0.07	$672.49	368.36	0.07	1.32	0.41	0.00	10.35%	0.06		0.08	
Nonmedical	-0.01	0.33	0.97	$-2.73	72.64	0.97	-0.04	0.29	0.88	-0.16%	0.01		0.88	
*Level of care (Ref: Pharmacy)*														
Primary	0.19	0.09	0.03	$55.93	25.65	0.03	0.18	0.09	0.05	0.89%	0.00		0.06	
Secondary	0.94	0.09	0.00	$408.90	45.78	0.00	0.94	0.10	0.00	7.16%	0.01		0.00	
Tertiary	1.30	0.15	0.00	$702.21	129.43	0.00	1.25	0.13	0.00	11.49%	0.02		0.00	
Number of inpatient nights	0.03	0.02	0.15	$15.02	10.19	0.15	0.03	0.02	0.20	0.21%	0.00		0.20	
Number of outpatient visits	0.00	0.00	0.89	-$0.26	1.83	0.89	0.00	0.00	0.84	-0.01%	0.00		0.84	
Number of medications	0.01	0.00	0.00	$2.20	0.43	0.00	0.00	0.00	0.00	0.04%	0.00		0.00	
**Health determinants**														
Share HHM with chronic illness	0.37	0.14	0.01	$193.26	68.67	0.01	0.40	0.13	0.00	3.39%	0.01		0.00	
Share HHM with disability	0.27	0.30	0.38	$139.01	155.96	0.38	0.23	0.27	0.39	1.94%	0.02		0.39	
Share HHM in SRH < good	-0.01	0.10	0.90	-$6.02	49.36	0.90	-0.01	0.10	0.92	-0.09%	0.01		0.92	
Severity score	0.16	0.03	0.00	$81.52	15.18	0.00	0.15	0.03	0.00	1.23%	0.00		0.00	
Days lost to illness/injury	0.01	0.00	0.00	$2.54	0.45	0.00	0.00	0.00	0.00	0.03%	0.00		0.00	
*Number of HHM with disease (Ref: Communicable diseases)*														
NCDs	0.14	0.04	0.00	$72.95	19.76	0.00	0.12	0.03	0.00	1.01%	0.00		0.00	
MNNDs	-0.21	0.11	0.05	-$110.10	56.25	0.05	-0.16	0.11	0.17	-1.33%	0.01		0.18	
Injuries	0.28	0.07	0.00	$144.52	35.22	0.00	0.25	0.07	0.00	2.09%	0.01		0.00	
**Social determinants**														
Household size	-0.03	0.02	0.14	-$16.99	11.51	0.14	-0.06	0.02	0.02	-0.47%	0.00		0.02	
HoHH age	0.01	0.00	0.01	$3.44	1.33	0.01	0.01	0.00	0.01	0.06%	0.00		0.01	
HoHH gender	0.07	0.06	0.25	$37.64	32.96	0.26	0.08	0.06	0.22	0.69%	0.01		0.22	
*HoHH education (Ref: No education)*														
Primary	0.17	0.06	0.00	$93.19	34.44	0.01	0.18	0.06	0.00	1.56%	0.01		0.01	
Secondary	0.15	0.14	0.30	$80.58	77.57	0.30	0.17	0.15	0.26	1.46%	0.01		0.30	
Higher	0.05	0.16	0.77	$24.46	82.41	0.77	0.03	0.16	0.86	0.24%	0.01		0.86	
Other	-0.05	0.10	0.62	-$23.76	48.10	0.62	-0.04	0.11	0.72	-0.31%	0.01		0.72	
Employment ratio	-0.23	0.11	0.03	-$120.45	56.53	0.03	-0.27	0.11	0.02	-2.31%	0.01		0.02	
Share HHM over 60	0.03	0.12	0.81	$15.89	64.50	0.81	0.07	0.14	0.61	0.60%	0.01		0.62	
Share HHM under 5	0.21	0.20	0.30	$109.53	105.94	0.30	0.22	0.21	0.29	1.88%	0.02		0.28	
*Wealth quintile (Ref: Quintile 1)*														
Quintile 2	0.42	0.09	0.00	$134.16	30.36	0.00	-0.08	0.09	0.42	-0.77%	0.01		0.42	
Quintile 3	0.59	0.07	0.00	$207.22	27.04	0.00	-0.18	0.08	0.02	-1.72%	0.01		0.02	
Quintile 4	0.77	0.08	0.00	$297.39	39.05	0.00	-0.26	0.08	0.00	-2.40%	0.01		0.00	
Quintile 5	1.06	0.09	0.00	$485.53	51.41	0.00	-0.42	0.10	0.00	-3.54%	0.01		0.00	
Total indebtedness	0.00	0.00	0.94	$0.00	0.00	0.94	0.00	0.00	0.80	0.00%	0.00		0.80	
*Geographic domain (Ref: Capital)*														
Other urban	0.59	0.16	0.01	$318.00	112.43	0.01	0.64	0.17	0.00	5.56%	0.02		0.00	
Rural	0.40	0.08	0.00	$194.70	45.08	0.00	0.44	0.09	0.00	3.40%	0.01		0.00	
Constant	2.508	0.21	0.00				-5.11	0.23	0.00					

Abbreviations: HHM = household member; HoHH = head of household; MNNDs = maternal, neonatal, and nutritional diseases; NCDs = noncommunicable diseases; OOPE = out-of-pocket health expenditures; SE = standard error; SRH = self-reported health

Notes: 3,134 observations in both models

**Table 3:** GLM estimates of outpatient and inpatient OOPE (reference categories for inpatient model in brackets)

	OOPE for outpatient care						OOPE for inpatient care					
	Coef	SE	*p*-value	Marginal effect	SE	*p*-value	Coef	SE	*p*-value	Marginal effect	SE	*p*-value
**Healthcare determinants**												
*Sector of care (Ref: Public outpatient (Public inpatient))*												
Private outpatient (inpatient)	0.77	0.13	0.00	$271.50	44.84	0.00	0.71	0.14	0.00	$323.97	70.15	0.00
Overseas outpatient (inpatient)	0.60	0.92	0.51	$195.36	394.62	0.62	2.27	0.35	0.00	$2,722.00	976.03	0.01
Nonmedical outpatient	-0.09	0.31	0.77	-$19.95	67.74	0.77						
*Level of care (Ref: Pharmacy (Secondary for inpatient))*												
Primary	0.19	0.08	0.01	$54.33	21.15	0.01						
Secondary	1.13	0.09	0.00	$540.37	71.40	0.00						
Tertiary	1.38	0.22	0.00	$769.26	228.45	0.00	0.91	0.18	0.00	$610.90	165.44	0.00
Number of inpatient nights							0.16	0.02	0.00	$82.48	12.33	0.00
Number of outpatient visits	0.00	0.00	0.70	-$0.61	1.57	0.70						
Number of medications	0.01	0.00	0.00	$2.26	0.45	0.00						
**Health determinants**												
Share HHM with chronic illness	0.47	0.17	0.01	$215.13	79.56	0.01	0.29	0.30	0.33	$155.29	158.06	0.33
Share HHM with disability	0.49	0.34	0.16	$223.08	158.65	0.16	0.33	0.49	0.50	$175.72	262.71	0.51
Share HHM in SRH < good	-0.11	0.10	0.29	-$48.31	46.04	0.30	-0.06	0.17	0.74	-$30.25	91.61	0.74
Severity score	0.18	0.03	0.00	$82.13	16.01	0.00	-0.01	0.04	0.76	-$5.66	18.53	0.76
Days lost to illness/injury	0.01	0.00	0.00	$2.85	0.45	0.00	0.01	0.00	0.00	$3.60	1.26	0.01
*Number of HHM with disease (Ref: Communicable diseases)*												
NCDs	0.14	0.04	0.00	$64.20	19.47	0.00	0.03	0.06	0.62	$16.70	33.44	0.62
MNNDs	-0.52	0.09	0.00	-$237.59	51.25	0.00	0.64	0.13	0.00	$338.94	80.73	0.00
Injuries	0.12	0.11	0.26	$55.39	48.15	0.25	0.59	0.10	0.00	$311.83	60.81	0.00
**Social determinants**												
Household size	-0.01	0.02	0.70	-$4.26	10.79	0.69	-0.07	0.03	0.02	-$38.88	15.96	0.02
HoHH age	0.01	0.00	0.03	$2.49	1.17	0.04	0.00	0.00	0.32	$2.35	2.34	0.32
HoHH gender	0.01	0.07	0.92	$3.46	32.34	0.92	0.11	0.12	0.34	$58.85	62.00	0.35
*HoHH education (Ref: No education)*												
Primary	0.20	0.07	0.00	$98.90	35.60	0.01	-0.26	0.10	0.01	-$122.51	44.38	0.01
Secondary	0.04	0.13	0.74	$19.91	61.17	0.75	0.23	0.16	0.15	$140.53	103.79	0.18
Higher	0.06	0.15	0.70	$25.40	68.57	0.71	-0.22	0.19	0.23	-$108.08	79.60	0.18
Other	-0.09	0.10	0.39	-$37.66	42.45	0.38	0.47	0.30	0.12	$323.21	249.69	0.20
Employment ratio	-0.12	0.11	0.27	-$53.56	48.98	0.28	-0.06	0.22	0.81	-$29.18	118.68	0.81
Share HHM over 60	0.19	0.14	0.19	$86.63	65.55	0.19	0.37	0.28	0.18	$196.67	144.34	0.18
Share HHM under 5	0.33	0.20	0.09	$153.05	92.33	0.10	-0.74	0.41	0.07	-$389.92	220.16	0.08
*Wealth quintile (Ref: Quintile 1)*												
Quintile 2	0.36	0.09	0.00	$105.02	25.21	0.00	0.79	0.19	0.00	$233.87	70.14	0.00
Quintile 3	0.52	0.08	0.00	$163.71	24.92	0.00	0.98	0.15	0.00	$325.31	56.17	0.00
Quintile 4	0.75	0.09	0.00	$269.55	41.77	0.00	0.98	0.15	0.00	$322.04	62.14	0.00
Quintile 5	0.94	0.10	0.00	$372.05	46.90	0.00	1.28	0.15	0.00	$504.90	60.07	0.00
Total indebtedness	0.00	0.00	0.85	$0.00	0.00	0.85	0.00	0.00	0.14	$0.00	0.00	0.12
*Geographic domain (Ref: Capital)*												
Other urban	0.59	0.20	0.01	$269.18	126.61	0.04	0.13	0.22	0.55	$81.55	138.74	0.56
Rural	0.47	0.09	0.00	$199.67	43.40	0.00	-0.11	0.13	0.38	-$60.71	70.92	0.39
Constant	2.49	0.20	0.00				3.45	0.36	0.00			

Abbreviations: HHM = household member; HoHH = head of household; MNNDs = maternal, neonatal, and nutritional diseases; NCDs = noncommunicable diseases; OOPE = out-of-pocket health expenditures; SE = standard error; SRH = self-reported health

Notes: 3,014 observations in the outpatient model and 702 in the inpatient model

### Shapley decomposition results

Table 4 details the results of the Shapley decomposition analysis, which quantified the contributions of *healthcare*, *health*, and *social* variables to the explained variation in our outcomes. *Healthcare* variables consistently accounted for the largest share of explained variation across outcomes, with contributions ranging from 41.36% for OOPE on inpatient care to 50.73% for outpatient OOPE. *Health* variables also contributed substantially across all outcomes. This was particularly true for the OOPE budget share (45.38%), where their contribution was nearly equal to that of *healthcare* variables. *Social* variables, while generally less influential across all outcomes, showed more pronounced contributions in inpatient OOPE at 33.02%, exceeding the contributions from *health* variables. For our three specifications, we note a considerable shift in explanatory power as more groups are added, with *healthcare* variables increasingly dominating, while the contributions of *social* and *health* variables to the explanatory power of the models diminish progressively for all outcomes (Table 7a to 7d, Additional file 1).

**Table 4:** Shapley decomposition results: Group contributions to the explained variance

	Total OOPE		OOPE budget share		Outpatient OOPE		Inpatient OOPE	
Group	%	95% CI	%	95% CI	%	95% CI	%	95% CI
Healthcare	46.41	42.56–50.24	45.96	42.61–49.19	50.73	45.38–55.47	41.36	23.63–50.85
Health	40.27	36.20–43.36	45.38	42.14–48.90	37.51	32.50–42.28	25.62	17.45–38.66
Social	13.31	10.73–16.72	8.59	6.66–10.88	11.76	9.12–15.82	33.02	22.41–50.62

Abbreviations: CI = confidence interval; OOPE = out-of-pocket health expenditures

Notes: Contributions are expressed relative to the total variance explained at the mean with 95% CIs

**Fig. 2:**
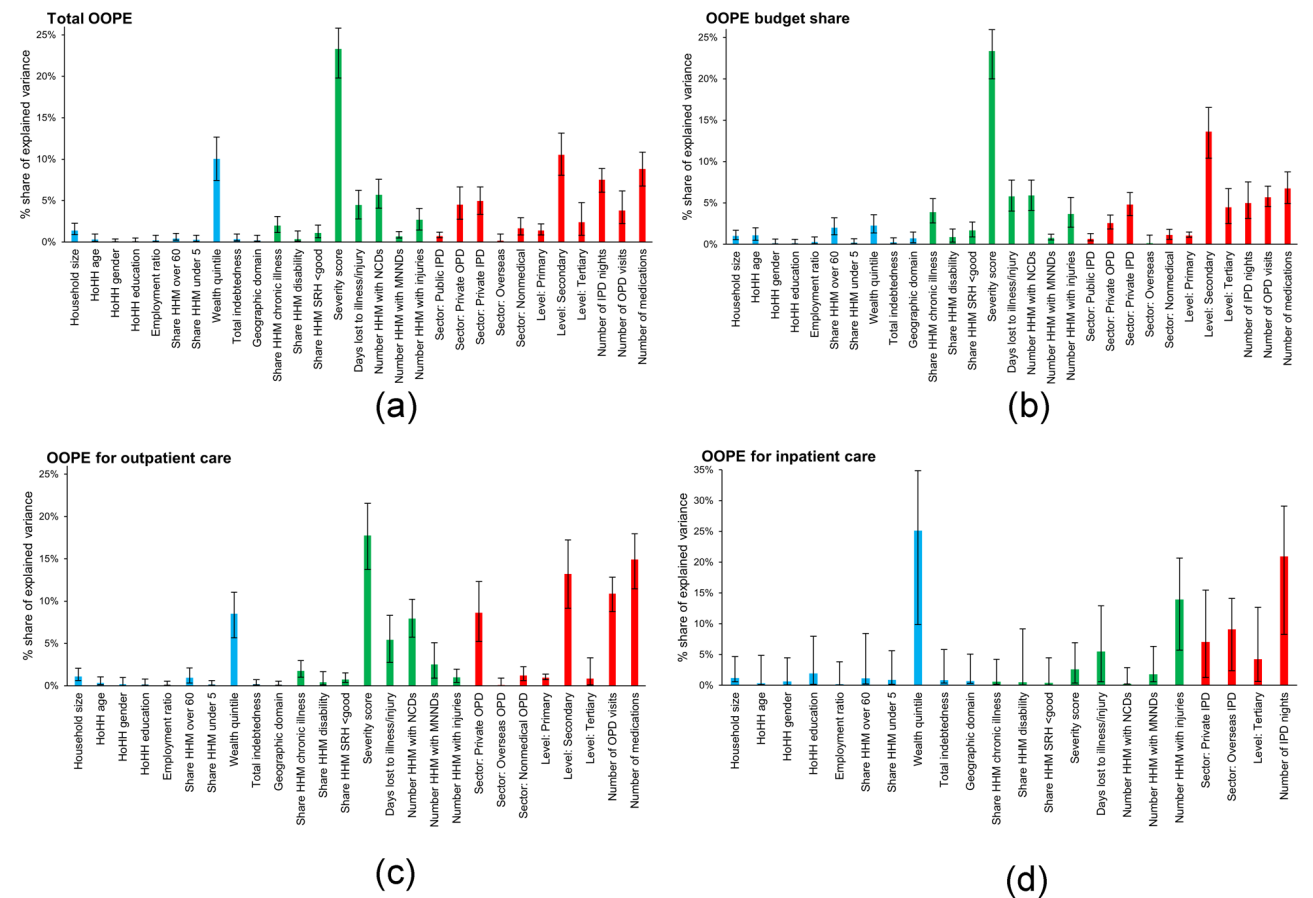
(**a**) Shapley decomposition results: *Healthcare*, *health*, and *social* contributions to the explained variance of total OOPE. Notes: Blue = social determinants; green = health determinants; red = healthcare determinants. The error bars represent the 95% confidence interval for each *healthcare*, *health*, and *social* variable. (**b**). Shapley decomposition results: *Healthcare*, *health*, and *social* contributions to the explained variance of the OOPE budget share. Notes: Blue = social determinants; green = health determinants; red = healthcare determinants. The error bars represent the 95% confidence interval for each *healthcare*, *health*, and *social* variable. (**c)**. Shapley decomposition results: *Healthcare*, *health*, and *social* contributions to the explained variance of outpatient OOPE. Notes: Blue = social determinants; green = health determinants; red = healthcare determinants. The error bars represent the 95% confidence interval for each *healthcare*, *health*, and *social* variable. (**d)** Shapley decomposition results: *Healthcare*, *health*, and *social* contributions to the explained variance of inpatient OOPE. Notes: Blue = social determinants; green = health determinants; red = healthcare determinants. The error bars represent the 95% confidence interval for each *healthcare*, *health*, and *social* variable

Figure 2a and d illustrate the individual contributions of explanatory variables and their 95% confidence intervals, with full numerical details for our three specifications and all four outcomes available in Tables 7a through 7d in the Additional file 1. Severity consistently emerged as the dominant variable contributing to total OOPE, the OOPE budget share, and outpatient OOPE, accounting for the largest share of explained variation among individual explanatory variables at 23.32%, 23.34%, and 17.75% for each outcome, respectively. Other notable *health* contributors included the number of household members with NCDs, with shares between 5.71% and 7.95%, and the number of days lost to illness/injury, ranging from 4.46% of the total OOPE to 5.80% of the budget share. Among the *healthcare* variables, secondary care and the number of medications were the most influential: secondary care accounted for 10.53–13.64% of the explained variance across these three models, while the number of medications contributed 6.47% to the explained variance in the OOPE budget share, 8.82% in total OOPE, and was particularly important for outpatient OOPE at 14.93%. Private sector care in both outpatient and inpatient settings also contributed substantially, varying from 7.73% in the budget share to 9.45% in the total OOPE model. Additional *healthcare* variables, such as the number of inpatient nights (4.99–7.53%) and outpatient visits (3.81–10.88%) also proved important, albeit less so. The wealth quintile was the only *social* variable to make considerable contributions to total OOPE (10.04%) and to OOPE for outpatient care (8.50%), though it contributed only 2.29% to the budget share.

In contrast to other outcome variables, our analysis of OOPE for inpatient care revealed distinct patterns. The number of inpatient nights led the *healthcare* contributions at 20.94%, followed by 9.12% for overseas care, 7.06% for private care, and 4.23% for tertiary care. Among the *health* variables, injuries and days lost to illness/injury were attributed the largest share of the explained variance at 13.96% and 5.47%, respectively. Notably, contrary to its dominance in the previous models, severity contributed only a minor share (2.61%) to the explained variance for inpatient OOPE. The wealth quintile was the predominant individual contributor at 25.14% of the explained variance, with minimal contributions from other *social* variables.

## Discussion

This study is the first to assess the *healthcare*, *health*, and *social* determinants of total OOPE, OOPE for outpatient and inpatient care, and the OOPE budget share, as well as their relative importance, within a population of households not covered by any prepayment scheme and engaged in informal employment. Our analysis of 3,254 households revealed that 96.76% sought outpatient care and 22.16% utilized inpatient care, with a vast majority (92.10%) visiting private providers. Nearly all households incurred OOPE, averaging $475.30 annually per household with a budget share of 7.84%. We observed high incidences of catastrophic and impoverishing spending and considerable reductions in food and other essential expenditure to manage OOPE, highlighting high levels of financial hardship in this population, which may have long-term detrimental effects on household welfare and social mobility. The determinants influencing our outcomes included *healthcare*, *health*, and *social* variables. *Healthcare* variables emerged as the most substantial group contributors across all outcomes, driven by the level of care, the use of private sector care, and the intensity of utilization (including the number of medications, outpatient visits, and inpatient nights). In terms of *health* determinants, chronic illness, perceived severity, days lost to illness/injury, and type of illness were significant, with severity being the single most substantial contributor to the explained variance in total OOPE, outpatient OOPE, and the OOPE budget share. Among the *social* variables, while several were significant, the wealth quintile was the predominant contributor to the explained variance across all outcome variables.

## Interpretation of findings

Among our three groups, *healthcare* variables consistently emerged as the dominant contributors to the explained variance across all outcomes in our Shapley decomposition analysis. This aligns with a study from India where healthcare factors accounted for 76.0% of the variance in the budget share [24].

Specifically, our analysis highlights the critical role of the private sector in explaining OOPE outcomes and the OOPE budget share, corroborating global studies [20, 28, 29, 31] and supporting data showing that 76.7% of Cambodia’s OOPE was channelled into private healthcare in 2016 [59]. From 2009 to 2023, the number of private providers in Cambodia surged to 21,842, including mostly pharmacies (3,747) and lower-level facilities (16,776), far outnumbering the 1,567 public facilities [53]. The preference for private services is largely driven by supply-side constraints in public facilities, including shortages of doctors, equipment, and medications, especially at lower-level facilities [60, 61]. This issue is exacerbated by the prevalence of ‘dual practice’, where over 50% of private healthcare workers also held public positions as of 2015, undermining the accessibility, efficiency, and perceived quality of public healthcare, and thereby further driving the demand for private care [60, 62, 63]. Informal workers, who typically rely on daily earnings, prefer private providers for their accessibility, shorter waiting times, and perceived responsiveness [60, 64, 65], even though our results indicate that this choice leads to higher OOPE.

The number of medications emerged as a significant determinant and strong contributor to the explained variance in our models, particularly for outpatient OOPE. This finding aligns with Cambodian data showing that 83.38% of OOPE were spent on medications, reflecting trends similar to those in other LMICs [1, 24, 66, 67]. With the rapid expansion of private providers and pharmacies over the past decade, there has been a notable increase in medication consumption in Cambodia, where these providers often charge considerably higher prices than international reference prices due to the lack of pricing regulations [65]. This situation is particularly challenging for our study population of informal workers and their dependents, who typically lack employment benefits such as paid sick leave [5]. These individuals frequently use medications to manage symptoms and shorten recovery periods, aiming to minimize income loss due to work absences. Additionally, the strong cultural preference for medication-based treatments in Cambodia further encourages higher medication consumption [68]. Remarkably, in the combined models for total OOPE and the OOPE budget share, medications were more influential than both the number of inpatient nights and the number of outpatient visits. This indicates that the type and intensity of treatment during healthcare visits, rather than the frequency of visits, are crucial in determining these outcomes. Studies from India and Bangladesh also reported that the private sector and medications purchased therein were the largest contributors to OOPE and the OOPE budget share [24, 29].

Higher levels of care were they largest *healthcare* contributors to the explained variance in total OOPE, the OOPE budget share, and outpatient OOPE. This corroborates findings from Zambia, where higher levels of care similarly increased OOPE [31]. In Cambodia, the primary healthcare system exhibits pronounced weaknesses in both the public and private sector [60]. Therefore, and in the absence of a gatekeeping and referral mechanism for uncovered households, many may opt for care at higher levels of the health system based on perceived quality rather than medical necessity—a trend that is common in many LMICs and often results in higher expenditures [69]. Importantly, among households seeking outpatient care, over 90% of secondary care utilization were visits to private clinics and hospitals, further underscoring the important role of private providers as determinants of our outcomes.

Among the *health* variables, the prevalence of chronic illness and the number of household members treated for NCDs significantly increased total OOPE, OOPE for outpatient care, and the OOPE budget share. This finding aligns with global studies [18, 19, 21–24, 35, 36, 70], global trends in lack of coverage for NCDs [71], and prior research in Cambodia showing that a substantial share of NCD treatment and care is paid out-of-pocket [72]. Conversely, injuries significantly elevated OOPE for inpatient but not for outpatient care, reflecting their acute nature, which often necessitates inpatient stays—a pattern similarly observed in India and Pakistan [24, 28].

Our Shapley decomposition analysis revealed that *health* determinants made substantial contributions to our outcomes, particularly to total OOPE and the OOPE budget share, accounting for 40.28% and 45.40% of the explained variance, respectively. Notably, perceived illness severity and days lost to illness/injury were strong contributors, which may stem from their multifaceted influence on healthcare decisions and expenditure patterns. Conditions that are (perceived as) more severe often necessitate multiple healthcare visits from multiple providers or specialists, require seeking services at higher levels of care, demand more extensive diagnostic testing, and involve costlier treatments and medications—all of which directly influence OOPE and the OOPE budget share. Additionally, the number of days lost to illness/injury reflect the economic consequences of severe and prolonged illnesses. In addition to contributing to higher direct medical costs, more severe conditions may also generate indirect costs, such as lost income for the sick person and their caregiver [73], increasing the overall financial burden on households. This finding concurs with results from India, where missed productive days contributed strongly to the budget share [24], and from Bangladesh, where perceived severity was the second most important variable driving OOPE [29]. The lower contribution of severity to inpatient OOPE suggests less variability in severity among households seeking care in inpatient settings, reducing its explanatory power in that model.

NCDs made notable contributions to outpatient OOPE, total OOPE, and the OOPE budget share. This likely reflects the chronic and complex nature of NCDs, which generally require regular monitoring, ongoing medication, frequent provider visits, and often the management of multiple comorbidities. This generates recurring expenses that cumulatively place a significant financial burden on households [71]. Additionally, NCDs typically affect adults in their working years, creating a dual burden where households simultaneously face reduced earning capacity and increased healthcare expenditures, thus raising the OOPE budget share. The substantial contribution of NCDs is particularly concerning giving the rising prevalence of these conditions in LMICs, where health systems are often poorly equipped to provide comprehensive NCD care [71]. In Cambodia, the public sector currently faces severe gaps in NCD management from primary to specialist care, driving patients to the private sector where NCD medications are more readily available but more expensive [74, 75]. Conversely, NCDs contributed minimally to OOPE for inpatient care, suggesting that they are predominantly managed in outpatient settings.

Among the *social* variables, increasing age and education level of the HoHH were associated with increased OOPE, reflecting findings from Bhutan, China, Pakistan, Zimbabwe, and Bangladesh [16, 17, 22, 28, 34–36]. Although increasing household size only significantly lowered inpatient OOPE and the OOPE budget share, it generally correlated with decreased OOPE across multiple studies [17, 19, 28, 29, 36]. This reduction could be attributed to intrahousehold risk-pooling, which mitigates the financial impact of OOPE in large households with more earning members, or their capacity for informal caregiving, which reduces the need for costly inpatient services [76, 77]. However, another perspective suggests that resources in larger households may be spread thinner, consequently reducing per capita OOPE [78]. Rural residents experienced higher OOPE and OOPE budget shares compared to those in the capital, supported by evidence from both Asian and African contexts [17, 20, 22, 24, 28, 30]. Geographic disparities in the organization and access to healthcare in Cambodia are pronounced, with the capital offering superior availability, accessibility, accommodation, and quality of care [60, 79]. Additionally, there are differences in the healthcare market between rural and urban areas. In rural areas, limited availability and competition among providers, coupled with the higher costs of service delivery, creates conditions where rural residents face higher OOPE and often additional indirect expenses, such as transportation. Furthermore, our findings indicated that the presence of young children in the household did not significantly increase OOPE outcomes, which is supported by a study from India [24]; however, other publications have reported contrary results [20, 21, 29, 35, 37]. Over the past few decades, Cambodia has prioritized investments in maternal and child health, leading to improvements in access to lower-cost services and corresponding health outcomes [41, 60]. Furthermore, wealthier households generally incurred higher OOPE, which is likely driven by higher disposable income and is consistent with the findings in numerous studies [17–19, 21, 27, 28, 30, 34, 36, 37]. However, the wealth gradient reversal for the OOPE budget share suggests that while absolute OOPE were higher, it constituted a smaller proportion of total household consumption expenditure for wealthier households, a pattern also observed in India [24].

Although many *social* variables were significant, Shapley decomposition showed that their contributions to the explained variance in our outcomes were modest. The notable exception was the wealth quintile, which emerged as a particularly strong contributor to inpatient OOPE, accounting for approximately 25% of the explained variance – comparable to the contributions of all *health* variables combined. This indicates that financial capacity plays a crucial role in the utilization of inpatient care, suggesting that poorer households may face financial barriers to accessing necessary inpatient services. The relatively modest contributions of other *social* variables in all models may be explained by several factors. First, our study population of nonpoor informal workers represents a relatively homogeneous socioeconomic group, which could reduce the explanatory power of *social* variables like education and employment. Second, the strong influence of *healthcare* and *health* variables might overshadow *social* factors in determining our outcomes—when faced with illness, households may prioritize obtaining necessary care regardless of their *social* characteristics. Third, some *social* variables may influence OOPE and the OOPE budget share indirectly through their effects on healthcare-seeking behaviour and health status, rather than directly affecting expenditure levels. For instance, education might influence health literacy and preventive behaviours, while household size might affect care-seeking decisions through resource pooling, but these indirect effects may not be fully captured in our analysis of direct contributions of these variables. Similarly, a study from India revealed that *social* characteristics, particularly driven by the wealth quintile and rural/urban status, contributed more strongly to inpatient OOPE than to other OOPE outcomes and the OOPE budget share [24].

Finally, our article has several limitations. All analyses are based on a single cross-sectional survey, precluding the comparison of any trends in our findings. The data were collected based on retrospective self-reports from heads of household, which, while generally reliable for OOPE data, can introduce bias in reporting illness and utilization information. Moreover, data on the number of medications were only available for outpatient care, potentially underestimating their relationship with total OOPE and the OOPE budget share. The study included only households where care was sought, which could lead to sample selectivity issues that we were unable to address with techniques such as Heckman correction. Aggregating data from the visit to the household level also resulted in a loss of detail. Furthermore, the use of 30-day recall periods, adjusted with time neutral annualization factors, may overestimate OOPE, suggesting that our estimates might represent the upper range of actual OOPE [40], even though our OOPE values largely concur with prior data from the Cambodia Socio-Economic Survey. Due to limited data availability, our models lack supply-side factors such as the number of government hospitals, doctors, or beds per catchment area, which have been shown to affect financial protection in Vietnam and Sri Lanka [20, 80]. Our analysis of OOPE for inpatient care was limited by a smaller sample size of 714 observations. Finally, all relationships reported in this study are associative and do not imply causality.

## Implications for policy and research

Our findings suggest that *healthcare* characteristics such as the sector and level of care, along with the intensity of utilization, largely drive the explained variation in our OOPE outcomes and the OOPE budget share. These determinants are directly addressable through public policy, highlighting the government’s important role in addressing OOPE and enhancing financial protection for uncovered households [24]. Given this, we propose several policy considerations. Combined with further research, these measures could advance equity and accelerate Cambodia’s progress toward UHC.

The high OOPE and budget shares as well as substantial gaps in financial protection highlighted by our study emphasizes the need for the Cambodian government to extend formal prepayment coverage to uncovered households. Given the challenges with expanding contributory schemes to informal workers [2, 4, 11], non-contributory mechanisms funded through public revenues—similar to the recent expansion of the Cambodian HEF to include additional near-poor households [81]—may be more feasible. Additionally, considering Cambodia’s fiscal challenges with high informality, it may be necessary to reassess and potentially reform how existing budget revenues are allocated to ensure more efficient resource use [4, 79, 82].

Given the demonstrated preference of uncovered households for private healthcare, the private sector appears vital for advancing UHC in Cambodia. Strengthening public healthcare alone may not sufficiently improve access or reduce OOPE for this group. Strategies to leverage private sector capacity that the Cambodian government may consider include forming public-private partnerships, particularly for essential services currently inadequately provided by the public sector, such as treatment for major NCDs like diabetes, hypertension, or cardiovascular diseases. These partnerships should be supported by a formal accreditation system to ensure private facilities meet defined quality standards. Additionally, it may be necessary to strengthen regulations on private sector practices, including around dual practice. Furthermore, the government could explore contracting with private pharmacies under expanded prepayment schemes to enhance access to essential medicines at regulated prices. This approach is supported by our data showing that 94.43% of uncovered households purchased medications in the 30 days preceding the survey, with 60.42% doing so from private pharmacies [83]. As prepayment schemes are progressively expanded, the government might also consider implementing price controls based on international reference pricing to make essential medicines more affordable to the broader population, especially since medicine prices in the private sector are significantly higher than international reference prices [65]. Efforts to enhance affordable access to essential medicines may need to be supported by supply-side interventions such as enforcing medicine sale regulations, promoting rational prescribing and dispensing, enhancing quality controls, and improving consumer understanding about medication necessity and risks [84–87].

Our findings also support the need to scale up access to comprehensive management for NCDs and other chronic illnesses, which aligns with previous research in Cambodia [60, 74]. Notably, interventions aimed at prevention, early detection, and management could play an important role in mitigating severe episodes of these diseases and reducing the financial burden they impose on households.

Moreover, our results imply the need for an effective referral and gatekeeping system to prevent the unnecessary utilization of higher-level, more expensive healthcare services, which strongly contributed to the explained variance in OOPE and the OOPE budget share. Strengthening primary healthcare as the first point of contact, potentially in collaboration with the private sector, could ensure that services are delivered at the most appropriate level, aligning with Cambodia’s goal of bolstering primary healthcare [41, 88]. Implementation of any referral and/or gatekeeping system should occur alongside the gradual expansion of the health system’s capacities, ensuring reliable access to needed services at the appropriate levels of care.

Further research is required to show how other *health* determinants such as severity, days lost to illness/injury and injuries can be influenced by public policy. Additionally, our findings emphasize the need to expand research on the determinants of OOPE and the OOPE budget share among informal workers, a key demographic for advancing UHC that remains underexamined. Current research also largely focuses on *social* determinants, neglecting the importance of *health* and *healthcare* variables, which our study identified as dominant contributors to the explained variance in OOPE outcomes and the budget share. Future research should also employ longitudinal panel data to capture trends and changes over time. Finally, moving beyond average effects and examining how the relative importance of various determinants varies across different distribution points of continuous outcome measures such as OOPE and the OOPE budget share could provide nuanced findings important for targeted policies.

## Conclusion

In this study, we analysed the *healthcare*, *health*, and *social* determinants of OOPE and the OOPE budget share in Cambodia, focusing on a critical yet under-researched demographic for UHC—nonpoor informal workers and their dependents without formal coverage under any prepayment scheme. This study advances existing research by employing Shapley decomposition analysis, a method that in addition to identifying significant associations quantifies the relative contributions of these determinants. Our results highlight the dominant influence of *healthcare* determinants – and to a lesser extent, *health* determinants – in explaining variance in our outcomes, with the wealth quintile emerging as a notable *social* contributor.

Our findings underscore the need to integrate nonpoor informal workers and their dependents into formal prepayment schemes in Cambodia. We recommend that the government strategically engages with private providers and pharmacies. This could involve forming public-private partnerships for essential health services and contracting with private pharmacies under expanded prepayment schemes to enhance access to affordable medicines. Additionally, implementing an effective referral and gatekeeping system may help encourage utilization at the appropriate level of care. These measures are essential not only for reducing financial hardship among currently uncovered households, but also for advancing Cambodia’s broader health financing goals of reducing reliance on OOPE and for progressing toward UHC. While focused on Cambodia, our results contribute to the global evidence base on the determinants of OOPE and the OOPE budget share and offer insights for other LMICs with similar contexts that strive to improve financial protection for their nonpoor informal workers and their dependents. Additionally, the use of Shapley decomposition to quantify the relative importance of *healthcare*, *health*, and *social* determinants offers a robust analytical approach that can be adapted by other countries seeking to inform their public policy decisions around reducing OOPE and enhancing financial protection.

## Electronic supplementary material

Below is the link to the electronic supplementary material.

Supplementary Material 1

Supplementary Material 2

## Data Availability

The data that support the findings of this study are available from the Government of the Kingdom of Cambodia, but restrictions apply to the availability of these data, which were used under license for the current study, and so are not publicly available. However, the data are available from the authors upon reasonable request and with the permission of the Government of the Kingdom of Cambodia.

## Abbreviations

HHM: Household member
HoHH: Head of household
LMIC: Low- and middle-income country
MNND: Maternal, neonatal, nutritional disease
NCD: Noncommunicable disease
OOPE: Out-of-pocket health expenditures
UHC: Universal health coverage
$: United States dollar
