# Supplementary material for: Assessing the determinants of out-of-pocket health expenditures among Cambodian households in informal employment using survey data
Source: Int J Equity Health. 2025 Jan 31;24:33. doi: 10.1186/s12939-025-02394-6 (PMC11783865; doi:10.1186/s12939-025-02394-6)
Supplement: Supplementary file 1 — Supplementary Material 1 [file 12939_2025_2394_MOESM1_ESM.pdf]

Additional file 1

Contents

Household sampling and data collection ... 2

Sample size calculations ... 2

Sample selection ... 2

Data collection ... 3

Table 1. Outcome variables and their measurement ... 4

Table 2. Explanatory variables, their measurement, and expected direction of association with outcomes ... 5

Table 3a. Grouping of healthcare providers for outpatient care ... 7

Table 3b. Grouping of healthcare providers for inpatient care ... 7

Table 4. Grouping of disease categories ... 8

Table 5. Concentration of OOPE across population percentiles (mean) ... 8

Table 6. Validation of our survey data with the 2021 and 2019 Cambodian Socio-Economic Survey data ... 9

Table 7a. Shapley decomposition results: Total OOPE ... 10

Table 7b. Shapley decomposition results: OOPE budget share ... 11

Table 7c. Shapley decomposition results: OOPE for outpatient care ... 12

Table 7d. Shapley decomposition results: OOPE for inpatient care ... 13

References ... 14

Household sampling and data collection

The following section describes our multi-stage clustered sampling design employed for selecting households included in our survey. Figure 1 illustrates the level of hierarchies of the household sampling approach:

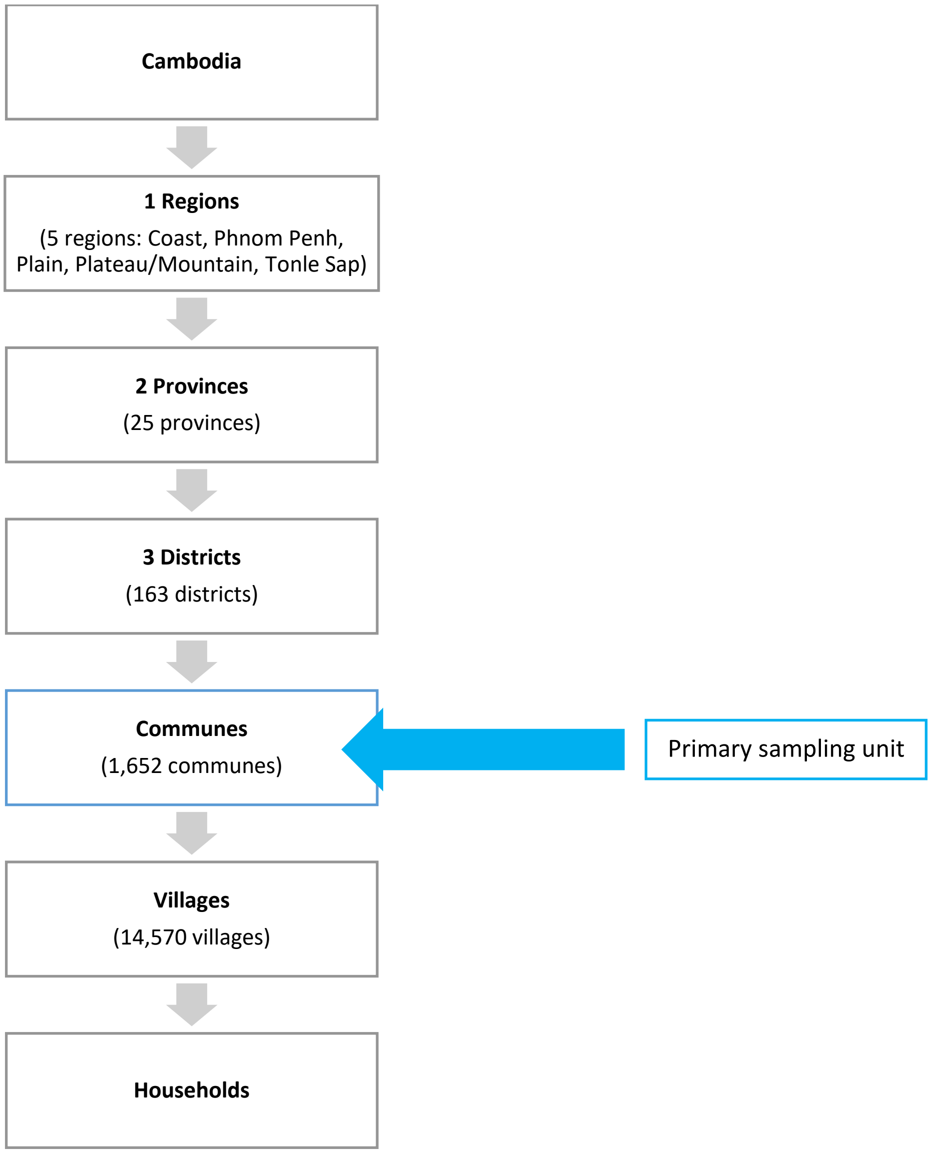

Figure 1. Outline of multi-stage cluster sampling procedure.

Sample size calculations

We performed detailed sample size calculations to ensure adequate representation of households incurring OOPE, targeting a margin of error of ±4.5%. This led to a minimum required sample size of 3,254 households distributed equally across 81 clusters (communes).

Sample selection

We first purposively selected seven provinces from each of the five geographic regions within Cambodia in accordance with the geographical decomposition defined for the Cambodia Socio-Economic Survey (CSES). Provinces were selected based on population concentration across regions. The chosen

provinces included Battambang and Siem Reap (Tonle Sap), Kampot (Coast), Kampong Speu and Kratie (Plateau/Mountain), Prey Veng (Plain), and the capital Phnom Penh (Phnom Penh). Jointly, these provinces represented approximately 46.28% of the Cambodian population.

Communes served as the primary clustering unit. The research team chose to cluster at the commune level, rather than at the village or district level, as based on previous research through the CSES and other studies, communes were believed to be the geographic unit at which the availability and type of health facilities varies in a meaningful way. To ensure equal representation across each province and account for district-level variations in healthcare provision and socioeconomic characteristics, one commune was randomly selected per district for a total of 75 communes; the remaining six communes were randomly distributed across the selected provinces. Finally, we selected additional communes in cases where we were not able to reach the required minimum sample size in the originally selected communes, resulting in a total of 102 surveyed communes.

Interviewers sought to survey households across all villages within the selected communes, with a target of 40 households per commune (cluster). Households were selected using a random walk method. The selection process began by calculating the required number of households per village, dividing the total number of villages in a commune by the required sample size for each commune. Subsequently, the total number of households in each village was divided by the target number of households per village. These calculations, based on CSES data, were verified by team supervisors at the commune offices. The resulting figure, 'n', was used as the interval between surveyed houses during enumeration, with teams stopping at every nth house. When village numbers exceeded the required sample, a lucky draw method was used to randomly select the necessary villages. In cases of high ineligibility rates or accessibility issues of villages, the random walk was repeated within the same village, skipping previously visited households and adjusting the interval 'n' as needed. In a few instances where villages had an excess of households and only 1 or 2 households required surveying, the enumeration team opted for an 'n' value of 25 and initiated the survey at a prominent landmark in the village. Household eligibility for the survey was determined by the following criteria:

i) No household member was currently or had been enrolled in any social health protection scheme in the past 12 months;
ii) At least one household member sought preventive, outpatient, or inpatient services within the defined recall periods.
iii) The of the household was an adult over the age of 18 and willing and able to provide verbal informed consent.

Data collection

Data collection was conducted by a Cambodian research firm over an eight-week period in June-July 2023. A team of 20 enumerators and 4 supervisors was recruited from their roster of experienced quantitative enumerators. Supervisors and enumerators underwent comprehensive training, including a detailed review of the survey questionnaire, mock interviews, ethical protocols, and practical use of the electronic data collection tool, SurveyCTO. Pilot testing over several days led to minor refinements of the survey tool. The research firm's field work manager conducted regular field observations to ensure all data collection protocols were being followed correctly. A full-time supervisor oversaw each of the enumeration teams, conducting repeat interviews with approximately 10% of respondents to verify survey responses. Additionally, the research team performed regular data quality back checks, periodically downloading and reviewing all data to ensure accuracy and proper survey functionality.

Table 1. Outcome variables and their measurement

Outcome variables	Measurement
Total OOPE	Annual household OOPE on consultation, diagnostic services, medications with and without prescription, herbs and traditional medicines, medical products, informal payments and gifts, food and hospital bed/room (inpatient care only), and other direct medical expenses at the point of service delivery for both outpatient and inpatient care
OOPE budget share	The share of total annualized household consumption expenditure (THCE) spent on total OOPE. THCE includes annual household consumption expenditure (monetary and in-kind) and the value of consumption of homemade products for five categories: food, non-food nondurables (including OOPE), education, housing, and durables.
OOPE for outpatient care	Similar to total OOPE, for outpatient care only (excludes food and hospital bed/room).
OOPE for inpatient care	Similar to total OOPE, for inpatient care only.

Table 2. Explanatory variables, their measurement, and expected direction of association with outcomes

Variable	Measurement	Expected direction of association			
		Total OOPE	Budget share	OOPE for outpatient	OOPE for inpatient
**Healthcare variables**					
Sector of care	The sector type accessed by any HHM.				
	1 = Public outpatient care (ref)				
	2 = Public inpatient care	+	+	+	+
	3 = Private outpatient care	+	+	+	+
	4 = Private inpatient care	+	+	+	+
	5 = Overseas	+	+	+	+
	6 = Nonmedical	-	-	-	-
Level of care	The highest level of care accessed by any HHM.				
	1 = Ancillary (pharmacies, ref)				
	2 = Primary	+	+	+	+
	3 = Secondary	+	+	+	+
	4 = Tertiary	+	+	+	+
No of inpatient nights	Annualized number of inpatient nights across HHM.	+	+	+	+
No of outpatient visits	Annualized number of outpatient visits across HHM.	+	+	+	+
No of medications	Annualized number of medications across HHM.	+	+	+	+
**Health variables**					
Share HHM with chronic illness	Share (%) of HHM who have a chronic illness. Chronic illness refers to any long-term or recurring condition requiring ongoing management, including medication, treatment, or other care, beyond just NCDs.	+	+	+	+
Share HHM with disability	Share (%) of HH members with a disability, generated based on the Washington Group short set of questions on functioning recommended cutoff. At least one domain is 'a lot of difficulty' or 'cannot do at all' [1].	+	+	+	+
Share HHM in self-reported health <good	Share (%) of HH members that report their health to be less than good. Self-reported health status was dichotomized into those who report their health status as "good" and "less than good" [2].	+	+	+	+
Severity score	Calculated as prevalence-weighed score for each household. Perceived severity score of each illness was multiplied by the number of household members affected by that illness and scores are then summed. Reflects both the severity and the prevalence of diseases within the household.	+	+	+	+

Days lost to illness/injury	Combined number of days HHM could not work due to inpatient or outpatient illness/injury.	+	+	+	+
Number of HHM with NCDs	Number of HHM who experienced an NCD. Defined based on IHME disease groupings [3].	+	+	+	+
Number of HHM with MNNDs	Number of HHM who experienced a MNND (including childbirth). Defined based on IHME disease groupings [3].	-	-	-	-
Number of HHM with injuries	Number of HHM who experienced an injury. Defined based on IHME disease groupings [3].	+	+	+	+
**Social variables**					
Household size	Household size in integers.	-	-	-	-
HoHH age	Age of HoHH in integers.	+	+	+	+
HoHH gender	0 = male (ref);<br>1 = female	 +	 +	 +	 +
HoHH education	0 = no formal education (ref)<br>1 = primary<br>2 = secondary<br>3 = higher<br>4 = other	 +<br>+<br>+<br>-	 +<br>+<br>+<br>-	 +<br>+<br>+<br>-	 +<br>+<br>+<br>-
Employment ratio	Share of household members in any employment, divided by the household size.	-	-	-	-
Share HHM over 60	Share (%) of HHM over the age of 60.	+	+	+	+
Share HHM under 5	Share (%) of HHM under the age of 5.	+	+	+	+
Wealth quintile	5 wealth quintiles based on total annual household consumption expenditure as a proxy of socio-economic status.<br>1 = Quintile 1 (ref); 2 = Quintile 2; 3 = Quintile 3; 4 = Quintile 4 ; 5 = Quintile 5	+	+	+	+
Total indebtedness	Total outstanding debt balance at the time of the survey interview.	-	-	-	-
Geographic domain	Residence of a household<br>1 = Phnom Penh<br>2 = Other Urban<br>3 = Rural	 <br>+<br>+	 <br>+<br>+	 <br>+<br>+	 <br>+<br>+

Abbreviations*:* HHM = household member; IHME = Institute of Health Metrics and Evaluation; MNND = maternal, neonatal, nutritional disease; NCD = noncommunicable disease; OOPE = out-of-pocket health expenditures.

Table 3a. Grouping of healthcare providers for outpatient care

Healthcare provider type	Sector	Level
Public Health Post	Public	Primary
Public health center	Public	Primary
Public district hospital	Public	Secondary
Public provincial hospital	Public	Secondary
Public national hospital	Public	Tertiary
Private pharmacy / dispensary	Private	Ancillary
Private consultation room	Private	Primary
Home/cabinet of doctor, nurse, or other trained private health worker	Private	Primary
Private clinic	Private	Secondary
Private medical analysis laboratory	Private	Ancillary
Private hospital (non-profit)	Private	Secondary
Private hospital (for-profit)	Private	Secondary
Treatment/care provided by a visiting provider at your home	Private	Primary
Shop selling drugs/market (includes also other stores such as grocery stores)	Non-medical	Primary
Traditional practitioner (e.g. Kru Khmer, Monk/religious leader, traditional birth attendant)	Non-medical	Primary
Overseas medical service	Overseas	Secondary

Table 3b. Grouping of healthcare providers for inpatient care

Healthcare provider type	Sector	Level
Public health center with bed	Public	Secondary
Public district hospital	Public	Secondary
Public provincial hospital	Public	Secondary
Public national hospital	Public	Tertiary
Home/cabinet of trained private health worker/nurse	Private	Secondary
Private clinic	Private	Secondary
Private hospital (non-profit)	Private	Secondary
Private hospital (for-profit)	Private	Secondary
Traditional practitioner (e.g. Kru Khmer, Monk/religious leader, traditional birth attendant)	Non-medical	Primary
Overseas medical service	Overseas	Tertiary

Table 4. Grouping of disease categories

Illness or injury categories in household questionnaire	Grouping for analysis
1 = Diabetes, blood, kidney, and other endocrine diseases	Non-communicable diseases
2 = Hypertension	Non-communicable diseases
3 = Cardiovascular diseases	Non-communicable diseases
4 = Cancers	Non-communicable diseases
5 = Mental health issues and substance use disorders	Non-communicable diseases
6 = Neurological disorders	Non-communicable diseases
7 = Other chronic disease - *Please specify*	Non-communicable diseases
8 = Injury/accident	Injuries
9 = Acute digestive diseases	Communicable diseases
10 = Fever	Communicable diseases
11 = Malaria, dengue, chikungunya, other tropical diseases	Communicable diseases
12 = COVID-19	Communicable diseases
13 = Acute respiratory disease	Communicable diseases
14 = TB, HIV/AIDS, Hepatitis	Communicable diseases
15 = Musculoskeletal disorders	Non-communicable diseases
16 = Skin diseases	Non-communicable diseases
17 = Maternal disorders	Maternal, neonatal, and nutritional diseases
18 = Neonatal disorders	Maternal, neonatal, and nutritional diseases
19 = Oral health conditions	Non-communicable diseases
20 = Childbirth / delivery	Maternal, neonatal, and nutritional diseases
888 = Other - Please specify	Regrouped into categories 1-20

Table 5. Concentration of OOPE across population percentiles (mean)

	Percentile					
	**Top 1%**	**Top 5%**	**Top 10%**	**Top 15%**	**Top 20%**	**Top 50%**
Share of total OOPE	20.56%	42.11%	56.50%	66.24%	73.41%	93.32%

Abbreviations: OOPE = out-of-pocket health expenditures.

Table 6. Validation of our survey data with the 2021 and 2019 Cambodian Socio-Economic Survey data

	Our survey	CSES 2023	CSES 2021
Number of households (at least 1 HHM sought care)	3,254	5,054	3,879
**Demographic characteristics**			
Household size	3.95	4.28	4.28
Age of head of household	46.75	49.33	49.46
Share HoHH who is female	35.56%	21.28%	19.82%
At least 1 person <5	36.96%	33.94%	33.25%
At least 1 person >60	27.11%	37.03%	37.26%
Share primary education only	57.40%	60.50%	61.70%
**Health characteristics**			
At least 1 HHM with chronic illness	50.32%	47.50%	33.63%
**THCE**			
Mean	$5,146	$6,300	$5,561
Quintile 1	$2,361	$3,689	$3,195
Quintile 2	$3,780	$4,740	$4,073
Quintile 3	$5,024	$5,651	$4,927
Quintile 4	$6,634	$6,391	$5,732
Quintile 5	$11,634	$10,617	$9,171
**OOPE**			
Mean	$475	$536	$463
Quintile 1	$187	$205	$166
Quintile 2	$295	$318	$253
Quintile 3	$362	$433	$352
Quintile 4	$504	$505	$464
Quintile 5	$1,016	$1,165	$988
**Financial protection indicators**			
Mean OOPE budget share	7.84%	7.23%	7.03%
Incidence of catastrophic OOPE (10%)	24.24%	23.89%	20.82%
Incidence of catastrophic OOPE (25%)	5.98%	6.43%	6.20%
Incidence of impoverishment (NPL)	6.67%	6.03%	5.55%

Abbreviations*:* CATA = catastrophic health expenditure; HHM = household member; NPL = national poverty line; OOPE = out-of-pocket health expenditures; US$ = United States dollar.
Notes*:* All dollar values are reported in 2023 US$.

Table 7a. Shapley decomposition results: Total OOPE

	Specification 1		Specification 2		Specification 3	
	%	95% CI	%	95% CI	%	95% CI
**Social determinants**	**100.00**	**-**	**20.944**	**17.02 - 25.36**	**13.751**	**10.73 - 16.72**
Household size	14.405	8.94 - 21.31	2.346	1.45 - 3.73	1.380	0.88 - 2.24
HoHH age	2.338	0.45 - 6.24	0.389	0.12 - 1.29	0.312	0.09 - 0.97
HoHH gender	0.169	0.09 - 2.07	0.065	0.04 - 0.49	0.041	0.02 - 0.37
HoHH education	0.177	0.13 - 1.33	0.184	0.08 - 0.71	0.095	0.05 - 0.48
Employment ratio	2.290	0.48 - 6.61	0.356	0.11 - 1.16	0.207	0.07 - 0.80
Share HHM over 60	4.176	1.02 - 9.03	0.648	0.37 - 1.52	0.400	0.23 - 1.04
Share HHM under 5	2.091	0.38 - 5.71	0.434	0.10 - 1.25	0.271	0.08 - 0.81
Wealth quintile	70.206	59.27 - 76.49	15.311	11.54 - 19.07	10.043	7.41 - 12.67
Total indebtedness	3.466	1.02 - 7.87	0.587	0.22 - 1.59	0.352	0.14 - 0.97
Geographic domain	0.683	0.44 - 2.83	0.155	0.11 - 0.69	0.212	0.10 - 0.83
**Health determinants**			**79.056**	**74.64 - 82.99**	**40.039**	**36.20 - 43.36**
Share HHM with chronic illness			3.735	2.37 - 5.65	1.997	1.15 - 3.07
Share HHM with disability			0.615	0.20 - 1.96	0.339	0.10 - 1.32
Share HHM in SRH <good			2.384	1.21 - 3.98	1.081	0.50 - 2.03
Severity score (log)			47.510	42.19 - 51.50	23.321	19.80 - 25.81
Days lost to illness/injury (log)			8.748	5.68 - 11.69	4.456	2.80 - 6.25
Number of HHM with disease (Ref: Communicable diseases)						
NCDs			10.549	7.92 - 13.47	5.711	4.10 - 7.57
MNNDs			1.435	0.71 - 2.66	0.694	0.47 - 1.24
Injuries			4.551	2.66 - 6.70	2.676	1.43 - 4.04
**Healthcare determinants**					**46.311**	**42.56 - 50.24**
Sector of care (Ref: Public outpatient care)						
Public inpatient care					0.724	0.50 - 1.18
Private outpatient care					4.503	2.76 - 6.65
Private inpatient care					4.947	3.36 - 6.66
Overseas					0.161	0.02 - 0.94
Nonmedical					1.630	0.86 - 2.93
Level of care (Ref: Pharmacy)						
Primary					1.370	0.87 - 2.19
Secondary					10.526	8.04 - 13.14
Tertiary					2.390	0.81 - 4.74
Number of inpatient nights					7.534	6.02 - 8.89
Number of outpatient visits					3.814	2.24 - 6.17
Number of medications					8.815	6.74 - 10.84
**R2**	0.09		0.30		0.32	

Abbreviations: CI = confidence interval; HHM = household member; HoHH = head of household; MNNDs = maternal, neonatal, and nutritional diseases; NCDs = noncommunicable diseases; OOPE = out-of-pocket health expenditures; SE = standard error; SRH = self-reported health.

Notes*:* 3,134 observations across all specifications. Outcome transformed with the natural log.

Table 7b. Shapley decomposition results: OOPE budget share

	Specification 1		Specification 2		Specification 3	
	%	95% CI	%	95% CI	%	95% CI
**Social determinants**	**100.00**	**-**	**8.92**	**6.37 - 11.61**	**8.59**	**6.66 - 10.88**
Household size	1.03	0.52 - 6.59	0.88	0.52 - 1.57	1.04	0.57 - 1.72
HoHH age	21.35	10.36 - 32.62	1.31	0.57 - 2.56	1.09	0.50 - 1.99
HoHH gender	4.15	0.32 - 14.31	0.22	0.04 - 0.95	0.16	0.04 - 0.66
HoHH education	2.75	0.39 - 12.48	0.15	0.06 - 0.70	0.13	0.06 - 0.62
Employment ratio	8.61	1.23 - 20.12	0.51	0.09 - 1.47	0.31	0.07 - 0.90
Share HHM over 60	49.53	29.47 - 61.42	2.75	1.41 - 4.41	2.00	1.15 - 3.21
Share HHM under 5	4.30	0.72 - 12.85	0.30	0.05 - 1.07	0.20	0.08 - 0.68
Wealth quintile	1.09	0.47 - 7.44	1.42	0.75 - 2.59	2.29	1.36 - 3.58
Total indebtedness	0.17	0.11 - 4.91	0.21	0.09 - 0.80	0.26	0.11 - 0.79
Geographic domain	7.01	1.19 - 17.61	0.62	0.09 - 1.52	0.71	0.18 - 1.49
**Health determinants**			**91.08**	**88.39 - 93.66**	**45.38**	**42.14 - 48.90**
Share HHM with chronic illness			6.45	4.52 - 8.83	3.90	2.57 - 5.52
Share HHM with disability			1.38	0.55 - 2.92	0.86	0.29 - 1.84
Share HHM in SRH <good			3.41	2.02 - 5.24	1.67	0.91 - 2.68
Severity score (log)			49.27	43.95 - 53.64	23.34	20.01 - 25.96
Days lost to illness/injury (log)			11.87	8.65 - 15.42	5.80	4.03 - 7.78
Number of HHM with disease (Ref: Communicable diseases)						
NCDs			11.18	8.34 - 14.23	5.90	4.10 - 7.75
MNNDs			1.62	0.87 - 2.84	0.75	0.55 - 1.23
Injuries			6.44	3.97 - 9.23	3.66	2.09 - 5.67
**Healthcare determinants**					**45.96**	**42.61 - 49.19**
Sector of care (Ref: Public outpatient care)						
Public inpatient care					0.65	0.41 - 1.29
Private outpatient care					2.56	1.86 - 3.53
Private inpatient care					4.81	3.48 - 6.26
Overseas					0.16	0.02 - 1.13
Nonmedical					1.12	0.59 - 1.82
Level of care (Ref: Pharmacy)						
Primary					1.08	0.85 - 1.49
Secondary					13.64	10.40 - 16.55
Tertiary					4.47	2.52 - 6.74
Number of inpatient nights					4.99	3.15 - 7.56
Number of outpatient visits					5.70	4.58 - 7.02
Number of medications					6.74	4.94 - 8.76
**R2**	0.03		0.32		0.39	

Abbreviations: CI = confidence interval; HHM = household member; HoHH = head of household; MNNDs = maternal, neonatal, and nutritional diseases; NCDs = noncommunicable diseases; OOPE = out-of-pocket health expenditures; SE = standard error; SRH = self-reported health.

Notes*:* 3,134 observations across all specifications. Outcome transformed with the natural log.

Table 7c. Shapley decomposition results: OOPE for outpatient care

	Specification 1		Specification 2		Specification 3	
	**%**	**95% CI**	**%**	**95% CI**	**%**	**95% CI**
**Social determinants**	**100.00**	**-**	**21.15**	**16.74 - 28.24**	**11.76**	**9.12 - 15.82**
Household size	12.58	6.53 - 20.57	2.16	1.11 - 4.09	1.11	0.61 - 2.07
HoHH age	3.80	0.99 - 9.03	0.59	0.24 - 1.60	0.34	0.15 - 1.05
HoHH gender	0.50	0.10 - 4.10	0.32	0.05 - 1.56	0.20	0.03 - 0.96
HoHH education	1.32	0.18 - 6.03	0.22	0.08 - 1.10	0.14	0.04 - 0.80
Employment ratio	0.83	0.24 - 4.36	0.14	0.08 - 0.91	0.08	0.05 - 0.55
Share HHM over 60	8.92	3.00 - 15.81	1.49	0.51 - 3.41	0.95	0.31 - 2.12
Share HHM under 5	0.31	0.23 - 2.95	0.23	0.11 - 1.02	0.14	0.07 - 0.62
Wealth quintile	68.89	55.88 - 75.65	15.47	10.86 - 20.07	8.50	5.66 - 11.05
Total indebtedness	1.94	0.57 - 6.67	0.33	0.16 - 1.30	0.18	0.09 - 0.71
Geographic domain	0.90	0.42 - 3.56	0.21	0.10 - 1.00	0.12	0.08 - 0.53
**Health determinants**			**78.85**	**71.79 - 83.28**	**37.51**	**32.50 - 42.28**
Share HHM with chronic illness			3.72	2.28 - 5.75	1.77	1.00 - 2.99
Share HHM with disability			0.64	0.19 - 2.53	0.39	0.08 - 1.68
Share HHM in SRH <good			1.82	0.74 - 3.61	0.72	0.38 - 1.52
Severity score (log)			40.32	32.96 - 46.06	17.75	13.75 - 21.56
Days lost to illness/injury (log)			10.35	5.94 - 14.82	5.44	2.77 - 8.31
Number of HHM with disease (Ref: Communicable diseases)						
NCDs			15.61	11.55 - 19.42	7.95	5.76 - 10.20
MNNDs			4.82	2.10 - 9.06	2.51	0.89 - 5.09
Injuries			1.57	0.64 - 3.22	0.98	0.39 - 1.96
**Healthcare determinants**					**50.73**	**45.38 - 55.47**
Sector of care (Ref: Public outpatient care)						
Private outpatient care					8.62	5.22 - 12.32
Overseas outpatient care					0.08	0.00 - 0.91
Nonmedical outpatient care					1.22	0.60 - 2.24
Level of care (Ref: Pharmacy)						
Primary					0.94	0.73 - 1.37
Secondary					13.23	9.16 - 17.23
Tertiary					0.84	0.11 - 3.28
Number of outpatient visits					10.88	8.76 - 12.84
Number of medications					14.93	11.44 - 17.95
**R2**	0.07		0.22		0.31	

Abbreviations: CDs = communicable diseases; CI = confidence interval; HHM = household member; HoHH = head of household; MNNDs = maternal, neonatal, and nutritional diseases; NCDs = noncommunicable diseases; OOPE = out-of-pocket health expenditures; SE = standard error; SRH = self-reported health.

Notes*:* 3,014 observations across all specifications. Outcome transformed with the natural log.

Table 7d. Shapley decomposition results: OOPE for inpatient care

	Specification 1		Specification 2		Specification 3	
	%	95% CI	%	95% CI	%	95% CI
**Social determinants**	**100.00**	**-**	**51.578**	**35.07 - 69.97**	**33.023**	**22.41 - 50.62**
Household size	4.547	1.69 - 16.17	2.007	0.81 - 8.63	1.173	0.55 - 4.67
HoHH age	0.310	0.25 - 9.72	0.321	0.17 - 5.13	0.363	0.14 - 4.88
HoHH gender	1.643	0.11 - 12.77	0.918	0.07 - 6.20	0.625	0.06 - 4.45
HoHH education	6.672	0.26 - 21.39	4.804	0.25 - 13.75	1.955	0.16 - 7.97
Employment ratio	2.768	0.16 - 19.89	0.914	0.13 - 7.59	0.182	0.09 - 3.82
Share HHM over 60	0.690	0.33 - 16.40	1.094	0.26 - 10.02	1.156	0.23 - 8.42
Share HHM under 5	2.829	0.36 - 15.59	0.888	0.22 - 6.95	0.906	0.19 - 5.64
Wealth quintile	77.860	41.44 - 79.60	39.239	15.58 - 50.07	25.143	9.87 - 34.88
Total indebtedness	1.179	0.66 - 12.27	0.700	0.35 - 8.20	0.829	0.37 - 5.84
Geographic domain	1.502	0.67 - 14.19	0.692	0.38 - 8.66	0.693	0.32 - 5.09
**Health determinants**			**48.423**	**30.06 - 64.94**	**25.619**	**17.45 - 38.66**
Share HHM with chronic illness			1.407	0.23 - 7.53	0.611	0.17 - 4.22
Share HHM with disability			0.147	0.06 - 10.30	0.494	0.08 - 9.18
Share HHM in SRH <good			0.296	0.10 - 5.12	0.409	0.08 - 4.47
Severity score (log)			7.437	1.01 - 15.64	2.609	0.38 - 6.92
Days lost to illness/injury (log)			12.443	1.78 - 22.59	5.474	0.59 - 12.90
Number of HHM with disease (Ref: Communicable diseases)						
NCDs			0.793	0.25 - 5.71	0.252	0.20 - 2.88
MNNDs			0.959	0.39 - 6.73	1.811	0.57 - 6.34
Injuries			24.940	9.82 - 33.04	13.959	5.70 - 20.68
**Healthcare determinants**					**41.359**	**23.63 - 50.85**
Sector of care (Ref: Public inpatient care)						
Private inpatient care					7.063	1.28 - 15.47
Overseas inpatient care					9.117	2.37 - 14.13
Level of care (Ref: Secondary)						
Tertiary					4.234	0.63 - 12.68
Number of inpatient nights					20.944	8.27 - 29.12
**R2**	0.06		0.11		0.17	

Abbreviations: CI = confidence interval; HHM = household member; HoHH = head of household; MNNDs = maternal, neonatal, and nutritional diseases; NCDs = noncommunicable diseases; OOPE = out-of-pocket health expenditures; SE = standard error; SRH = self-reported health.

Notes*:* 702 observations across all specifications. Outcome transformed with the natural log.

References

1. Washington Group on Disability Statistics. WG Short Set on Functioning (WG-SS) Syntax [Internet]. 2024 [cited 2024 Jan 8]. Available from: https://www.washingtongroup-disability.com/analysis/wg-short-set-on-functioning-wg-ss-syntax/

2. O'Donnell O, van Doorslaer, Eddy Wagstaff A, Lindelow M. Analyzing Health Equity Using Household Survey Data: A Guide to Techniques and Their Implementation. Washington D.C.; 2008.

3. Roser M, Ritchie H, Spooner F. "Burden of Disease" [Internet]. 2021 [cited 2023 Sep 3]. Available from: https://ourworldindata.org/burden-of-disease
