# Supplementary material for: Assessing the determinants of out-of-pocket health expenditures among Cambodian households in informal employment using survey data
Source: Int J Equity Health. 2025 Jan 31;24:33. doi: 10.1186/s12939-025-02394-6 (PMC11783865; doi:10.1186/s12939-025-02394-6)
Supplement: Supplementary file 2 — Supplementary Material 2 [file 12939_2025_2394_MOESM2_ESM.pdf]

Additional file 2

Table 1. Literature review on the determinants of financial protection indicators in low- and middle-income countries

No	Author	Title	Year	Country	Outcome(s)	Determinants
1	Herberholz et al	Medical, transportation and spiritual out-of-pocket health expenditure on outpatient and inpatient visits in Bhutan	2021	Bhutan	OOPE OPD<br><br>OOPE IPD	**OOPE OPD**<br>Increased OOPE<br>- Age, THCE, rural areas (compared to urban)<br>- Functional disability<br>- Frequency of visits, length of stay<br>Decreased OOPE<br>- Education<br>- Going to public primary, going to public secondary<br>**OOPE IPD**<br>Increased OOPE<br>- Age<br>- Frequency of visits (2+)<br>- Length of stay
2	Khalid et al	Health services utilization and out-of pocket (OOP) expenditures in public and private facilities in Pakistan: an empirical analysis of the 2013–14 OOP health expenditure survey	2021	Pakistan	OOPE OPD<br><br>OOPE IPD	**OOPE OPD**<br>Increased OOPE<br>- Age, wealth quintile, rural<br>Decreased OOPE<br>- HH size<br>**OOPE IPD**<br>Increased OOPE<br>- Age, wealth quintile, rural<br>- Private sector, accident/injury<br>Decreased OOPE<br>- HH size
3	Thuong et al	Determinants of catastrophic health expenditure in Vietnam	2020	Vietnam	Catastrophic spending	Increased catastrophic spending<br>- Severe illness - strongest association<br>- Higher share of elderly people<br>- Higher incidence of inpatient and outpatient visits<br>- Older HoHH<br>- HoHH education level<br>- Private facilities interacted with severity - increases odds

						<u>Decreased catastrophic spending</u><br>- Higher income quintile, wealth gradient like in our study<br>- HoHH employed<br>- Increased HH size<br>- Urban areas<br>- No of hospitals per 1000 population, % of commune health stations with doctor
4	Van Minha et al	Financial burden of household out-of pocket health expenditure in Viet Nam: Findings from the National Living Standard Survey 2002-2010	2013	Vietnam	Catastrophic spending	<u>Decreased catastrophic spending</u><br>- Increased HH size<br><u>Increased catastrophic spending</u><br>- More elderly<br>- More children <6<br>- Rural areas<br>- Increasing quintile
5	Li et al	Factors affecting catastrophic health expenditure and impoverishment from medical expenses in China: policy implications of universal health insurance	2012	China	Catastrophic spending	<u>Decreased catastrophic spending</u><br>- Increasing wealth status<br>- Increasing HH size<br>- At least 1 young child<br><u>Increased catastrophic spending</u><br>- Female HoHH<br>- Elderly members<br>- Unemployed person<br>- little education<br>- Rural areas<br>- Chronic illness/NCD<br>- Hospitalization
6	Falconi et al	Determinants of catastrophic healthcare expenditure in Peru	2018	Peru	Catastrophic spending	<u>Increased catastrophic spending</u><br>- Chronic condition<br>- Rural areas<br>- Poorer wealth quintile<br>- smaller households<br>- Medical tests, surgery, and medication associated with greatest odds for catastrophic spending
7	Ramirez-Agudelo et al	What are the factors associated with catastrophic health expenditure in Colombia? A multi-level analysis	2023	Colombia	Catastrophic spending	<u>Decreased catastrophic spending</u><br>- Increased employment<br>- Increased HoHH education<br>- Increasing wealth status<br>- Larger HH size

						- Urban areas<br><u>Increased catastrophic spending</u><br>- Increased HOHH age<br>- Female HoHH<br>- At least 1 elderly
8	Ahmed et al	Catastrophic healthcare expenditure and impoverishment in tropical deltas: evidence from the Mekong Delta region	2018	Vietnam	Catastrophic spending	<u>Increased catastrophic spending</u><br>- Age HoHH<br>- Ethnicity<br>- HH size<br>- Environmental or economic shock past 5 years<br>- Private facility utilization<br>- Increasing wealth<br>- Elderly person in household
9	Khan et al	Catastrophic healthcare expenditure and poverty related to out-of-pocket payments for healthcare in Bangladesh—an estimation of financial risk protection of universal health coverage	2018	Bangladesh	Catastrophic spending	<u>Decreased catastrophic spending</u><br>- Urban areas<br><u>Increased catastrophic spending</u><br>- Female HoHH<br>- Increase education HoHH<br>- Elderly members<br>- HH with reproductive age woman
10	Giang et al	Is Health Insurance Associated with Health Service Utilization and Economic Burden of Non-Communicable Diseases on Households in Vietnam?	2020	Vietnam	Catastrophic spending for OPD and IPD	**OPD**<br>- No HHMs with NCDs<br>- Sex and age of HoHH<br>- Elderly members<br>- Lower quintile<br>**IPD**<br>- Higher levels of care<br>- More HHMs with NCDs<br>- Lower quintile<br>- Elderly people<br>- Female HoHH
11	Ghimire et al	Cumulative incidence, distribution, and determinants of catastrophic health expenditure in Nepal: results from the living standards survey	2018	Nepal	Catastrophic spending	<u>Decreased catastrophic spending</u><br>- Urban areas<br>- Educated HoHH<br><u>Increased catastrophic spending</u><br>- Elderly people<br>- Children<br>- Chronic illness

						- Acute illness & injury<br>- Higher ratio of illnesses<br>- 1 and 2 quintile
12	Zeng et al	Utilization of Health Care and Burden of Out-of Pocket Health Expenditure in Zimbabwe: Results from a National Household Survey	2018	Zimbabwe	OOPE<br><br>Catastrophic spending	**OOPE**<br>- Larger HH size<br>- Urban areas<br>- Higher wealth<br>- Increasing age<br>- Increasing education<br>**Catastrophic spending**<br>- Household size<br>- Wealth quintile<br>- Urban areas<br>- IPD care
13	Yazdi-Feyzabadi et al	Prevalence and intensity of catastrophic health care expenditures in Iran from 2008 to 2015: a study on Iranian household income and expenditure survey	2018	Iran	Catastrophic spending	<u>Increased catastrophic spending</u><br>- Elderly in HH<br>- Hospitalized person<br>- Household used OPD care<br>- Rural area
14	Edeh	Exploring dynamics in catastrophic health care expenditure in Nigeria	2022	Nigeria	Catastrophic spending	<u>Decreased catastrophic spending</u><br>- Richer quintile<br>- HHs larger than 5 members<br><u>Increased catastrophic spending</u><br>- Unemployed HoHH<br>- Elderly members
15	Mamun et al	The Determinants of Household Out-of-Pocket (OOP) Medical Expenditure in Rural Bangladesh	2018	Bangladesh	OOPE	- Selection of private healthcare provider<br>- Access to sanitation facilities<br>- HH size<br>- Gender and education of HoHH<br>- Number of children under 5
16	Sato	Catastrophic health expenditure and its determinants among Nigerian households	2022	Nigeria	OOPE<br><br>Catastrophic spending	**OOPE**<br>- Increased education decreases OOPE<br>- Increased spending with increased wealth<br>- Increased spending in rural areas<br>**Catastrophic spending**<br>- Education decreases catastrophic spending<br>- Increased wealth decreases catastrophic spending

17	Su et al	Catastrophic household expenditure for health care in a low-income society: a study from Nouna District, Burkina Faso	2006	Burkina Faso	OOPE	- Chronic illness in HH<br>- Average illness episodes per adult<br>- Number of treatment episodes<br>- Treatment through professional care<br>- HH quintile (increased risk for lower quintiles)
18	You et al	Determinants of Out-of-Pocket Health Expenditure in China. Analysis Using China Health and Nutrition Survey Data	2011	China	OOPE	- Above 65<br>- Education of HoHH<br>- chronic disease<br>- Urban area<br>- Household income
19	Rahman et al	Health-Related Financial Catastrophe, Inequality and Chronic Illness in Bangladesh	2013	Bangladesh	OOPE<br><br>Catastrophic spending	**OOPE**<br>- Chronic illness<br>- HH size<br>- Average illness per child and adult<br>- Healthcare-seeking behavior<br>- Higher education level of the HoHH<br>- Wealth quintile<br>**Catastrophic spending**<br>- Average number of illness increased RR<br>- Chronic illness increased RR<br>- Private outpatient facilities increased RR<br>- Hospitalization biggest risk factor<br>- Poorer wealth quintiles, decreased HH consumption expenditure
20	Malik et al	Socio-economic determinants of household out-of-pocket payments on healthcare in Pakistan		Pakistan	OOPE	Increased OOPE<br>- HH expenditure<br>- Literate HoHH and spouse<br>- Unsafe drinking water<br>- Unhygienic toilet facilities<br>- Larger distance from health facilities<br>- Elderly members and children<br>Decreased OOPE<br>- Gender HoHH (male)<br>- Professional occupation (white collar)

21	Hassan et al	The inequalities and determinants of Households' Distress Financing on Out-of-Pocket Health expenditure in Malaysia	2022	Malaysia	Distress financing	- Total of household members aged 65 years and older<br>- Total of household members aged less than five years<br>- Socio-economic status<br>- Total of household members who received IPD (12 months)<br>- Decreased risk for wealthier households
22	Mutyambizi et al	Incidence, socio-economic inequalities and determinants of catastrophic health expenditure and impoverishment for diabetes care in South Africa: a study at two public hospitals in Tshwane	2019	South Africa	Catastrophic spending	- Gender (female)<br>- Wealth quintile (lower increases risk)<br>- Children reduce risk
23	Masiye et al	Determinants of Healthcare Utilisation and Out-of-Pocket Payments in the Context of Free Public Primary Healthcare in Zambia	2016	Zambia	OOPE	<u>Increased OOPE</u><br>- Distance to health facilities<br>- Age in years<br>- Region of residence<br>- HH per capita expenditure<br>- Compared to health post: Tertiary/secondary hospital, District hospital, Public health center, Private/other health facility
24	Rout et al	Does public health system provide adequate financial risk protection to its clients? Out of pocket expenditure on inpatient care at secondary level public health institutions: Causes and determinants in an eastern Indian state	2018	India	OOPE	- Increased education<br>- Higher caste
25	Loganathan et al	Socio-demographic determinants of out-of-pocket health expenditure in a rural area of Wardha district of Maharashtra, India	2017	India	OOPE	- Caste type<br>- Type of family<br>- Occupation HoHH
26	Mahumud et al	Distribution and Determinants of Out-of-pocket Healthcare Expenditures in Bangladesh	2017	Bangladesh	OOPE	<u>Increased OOPE</u><br>- Age group<br>- Marital status<br>- Urban communities<br>- Richest quintile<br>- Higher education<br><u>Decreased OOPE</u>

						- Earning status<br>- Not receiving financial benefits
27	Nakovics et al	Determinants of healthcare seeking and out-of-pocket expenditures in a "free" healthcare system: evidence from rural Malawi	2020	Malawi	OOPE	<u>Increased OOPE</u><br>- Individuals aged 15-39<br>- Individuals who are the HoHH<br>- Individuals with chronic illness<br>- Individuals who had been hospitalized<br>- Individuals requiring more accompanying persons<br>- Individuals from higher SES strata<br>- Individuals living in urban areas
28	Getachew et al	Catastrophic health expenditure and associated factors among households of non community based health insurance districts, Ilubabor zone, Oromia regional state, southwest Ethiopia	2023	Ethiopia	Catastrophic spending	<u>Increased risk</u><br>- Larger HH size<br>- Low average daily income<br>- Medium distance to health facilities<br>- No ambulance service<br>- Chronic disease
29	Rail et al	Catastrophic health expenditure on chronic non-communicable diseases among elder population: A cross-sectional study from a sub-metropolitan city of Eastern Nepal	2022	Nepal	Catastrophic spending	<u>Increased risk</u><br>- Marital status of elderly<br>- Poverty status<br>- Cancer co-morbid state
30	Rahman et al	Forgone healthcare and financial burden due to out-of-pocket payments in Bangladesh: a multilevel analysis	2022	Bangladesh	OOPE<br><br>Catastrophic spending	**OOPE**<br>- Chronic illness<br>- HH size<br>- Consumption quintile<br>- Place of residence<br>- IPD and OPD care-seeking behavior<br>**Catastrophic spending**<br>- Widowed/separated<br>- Chronic illness<br>- Utilize public or private health services<br>- Richest quintile
31	Sharma et al	Catastrophic health care expenditure and impoverishment in Bhutan	2023	Bhutan	Catastrophic spending<br><br>Impoverishment	**Catastrophic and impoverishing spending**<br><u>Increased risk</u><br>- Poorer households<br>- HoHH unemployed<br>- HHs with elderly members

						- Rural areas<br>Reduced risk<br>- Female HoHH<br>- HHs with more children
32	Liliana et al	Catastrophic expenditure due to out-of pocket health payments and its determinants in Colombian households	2016	Colombia	Catastrophic spending	Reduced risk<br>- Region<br>- Nuclear families<br>- Composite families<br>Increased risk<br>- Children (5 or younger)<br>- Elderly (65 or older)<br>- Inpatient services<br>- Subsidized insurance only<br>- Lower income quintile<br>- Number of HH members working
33	Aregbeshola et al	Determinants of catastrophic health expenditure in Nigeria	2018	Nigeria	Catastrophic spending	- Age of household members<br>- Education of Hohh<br>- Gender of HoHH<br>- Location<br>- Geopolitical zone<br>- Work status of HoHH (unemployed)<br>- Health insurance status<br>- Private health facility<br>- Non-chronic illness
34	Bashir et al	Incidence and determinants of catastrophic health expenditures and impoverishment in Pakistan	2021	Pakistan	Catastrophic spending<br><br>Impoverishment	Increased risk<br>- Older age<br>- Increased income<br>Lower risk<br>- Increased education<br>- Female HoHH<br>- Larger HH size<br>- Older HH members<br>- Urban areas

35	Sharma et al	Out-of-pocket expenditure for hospitalization in Haryana State of India: Extent, determinants & financial risk protection	2017	India	Catastrophic spending	Increased catastrophic spending<br>- Males<br>- Scheduled castes/tribes<br>- Poorest quintile<br>- Insured<br>- Admitted for NCDs<br>- Private facility
36	Sheikh et al	Disease-specific distress healthcare financing and catastrophic out-of-pocket expenditure for hospitalization in Bangladesh	2022	Bangladesh	Catastrophic spending for IPD	Reduced catastrophic spending<br>- More working members<br>- Increased HH size<br>Increased catastrophic spending<br>- Chronic illness in household<br>- Private facilities<br>- NCDs (compared to CDs)<br>- Quintiles 1-4<br>- More hospitalized members
37	Ahmed et al	Assessing the incidence of catastrophic health expenditure and impoverishment from out-of-pocket payments and their determinants in Bangladesh: evidence from the nationwide Household Income and Expenditure Survey 2016	2022	Bangladesh	Catastrophic spending	Increased risk<br>- Higher educated HoHH<br>- Larger HHs<br>- Highest asset quintile<br>Reduced risk<br>- HHs with older members<br>- Chronic illness<br>- Private facility
38	Kastor et al	Disease-specific out-of-pocket and catastrophic health expenditure on hospitalization in India: Do Indian households face distress health financing?	2018	India	Catastrophic spending<br><br>Distress financing	Increased risk<br>- Urban residence<br>- Middle and rich quintiles<br>Reduced risk<br>- HoHH over 60<br>- Female HoHH<br>- HoHH higher education
39	Liu et al	Trends and determinants of catastrophic health expenditure in China 2010-2018: a national panel data analysis	2021	China	Catastrophic spending	Reduced risk<br>- Highschool and above education<br>- Larger HHs<br>Increased risk<br>- IPD care<br>- Older HoHH<br>- Older members

						- Chronic illness<br>- Lower quintiles
40	Kumara et al	Patterns and determinants of out-of-pocket health care expenditure in Sri Lanka: evidence from household surveys	2016	Sri Langka	OOPE	Increased OOPE<br>**Demand-side factors**<br>- Literate HoHH<br>- Estate and rural HHs<br>- Lower-income HHs<br>- More elderly members<br>- Number of pre-school children<br>- Number of school-age children<br>- Number of members with chronic illness<br>- Ayurveda and traditional care<br>**Supply-side factors**<br>- Shorter distance to private facility<br>- Larger number of doctors, including specialists<br>Reduced OOPE<br>**Supply-side factors**<br>- Shorter distance to government hospital<br>- Increased number of beds in government hospital<br>- Dentists employed in government hospital
41	Liu et al	Measurement and determinants of catastrophic health expenditure among elderly households in China using longitudinal data from the CHARLS	2021	China	Catastrophic spending	Reduced risk<br>- Highschool and above education<br>- Larger HHs<br>Increased risk<br>- Living with a spouse<br>- Wealth quintile<br>- IPD care within past 12 months<br>- OPD care within past 30 days<br>- Members with disability<br>- Chronic illness
42	Okedo-Alex et al	A review of the incidence and determinants of catastrophic health expenditure in Nigeria: Implications for universal health coverage	2019	Nigeria	Catastrophic spending	Increased risk<br>- Older HoHH<br>- Smaller HH size<br>- Larger HH size<br>- Elderly in HH<br>- Increased education<br>- Low educational status<br>- Patient as primary income earner

						- Poor HH<br>- Low social class<br>- Hospitalized member<br>- HH with non-chronic illness<br>- Taking loan<br>- Private facility<br><u>Reduced risk</u><br>- Employed HoHH<br>- Female HoHH<br>- Informal financing arrangements<br>- Private facility
43	Rasul et al	Determinants of health seeking behavior for chronic non-communicable diseases and related out-of-pocket expenditure: results from a cross-sectional survey in northern Bangladesh	2019	Bangladesh	OOPE	<u>Increased OOPE</u><br>- People who sought qualified professional care<br>- People suffering from a major chronic NCD<br>- Elderly (60 years old and above)<br>- The least poor
44	Serván-Mori et al	Out-of-pocket expenditure on medicines in Bangladesh: An analysis of the national household income and expenditure survey 2016-17	2022	Bangladesh	OOPE on medicines out of total household consumption expenditure	<u>Increased OOPE for medicines</u><br>- Age of HoHH<br>- Male HoHH<br>- HoHH never married<br>- Demographic dependence<br>- Disability index<br>- Use of health services<br>- Rural location<br>- Chronic illness: Common infections, Diabetes, Cancer, Cardiovascular diseases, Other chronic disease<br><u>Reduced OOPE for medicines</u><br>- HoHH working<br>- Equivalent adults

45	Mohsin et al	Understanding variation in catastrophic health expenditure from socio-ecological aspect: a systematic review	2024	LMICs	Catastrophic spending	**Interpersonal predictors**<br>- Age<br>- Sex<br>- Education<br>- Income<br>- Place of residence (rural-urban differences in availability/quality of care)<br>**Disease-specific factors**<br>- Type of illness<br>- Diseases that require IPD<br>- NCD, cancer, chronic disease<br>- Perceived severity of illness<br>**Relational factors**<br>- Nuclear families<br>- HH size<br>- HoHH characteristics (gender, employment, education)<br>- Elderly<br>- Members with chronic illness<br>- Families with children<br>- Membership in safety net<br>**Institutional factors**<br>- Physician fees<br>- OPD visits<br>- Cost of medicine<br>- Diagnostic costs<br>- Indirect costs: food, accommodation, transport<br>- Health-related income loss<br>- Type of provider: Private providers increase risk<br>- Type of care: IPD care<br>- Areas underserved by public providers/infrastructure<br>**Community predictors**<br>- Comorbidity<br>- Poverty<br>- Stigma<br>- Prevalence of natural disasters<br>**Policy level**<br>- Lack of regulation of the healthcare market

46	Njagi et al	Understanding variations in catastrophic health expenditure, its underlying determinants and impoverishment in Sub-Saharan African countries: a scoping review	2018	SSA	Catastrophic spending	- HH economic status<br>- HH size<br>- Elderly<br>- Children <5<br>- HH member sociodemographic characteristics (employment status, education, sex, age)<br>- Type of healthcare provider<br>- Type of illness<br>- Type of social health insurance<br>- Geographic location<br>- Distance to health facilities
47	Buigut et al	Catastrophic health expenditure and its determinants in Kenya slum communities	2015	Kenya	Catastrophic spending	- HoHH age, gender, education<br>- Membership in social safety net<br>- Adult earning member<br>- Number of children<br>- IPD care<br>- Members with chronic disease<br>- Private facilities
48	Li et al	Multimorbidity and catastrophic health expenditure: Evidence from the China Health and Retirement Longitudinal Study	2022	China	Catastrophic spending	- Income<br>- Age<br>- Sex<br>- Education<br>- Employment status (unemployed, retired)<br>- Chronic illness<br>- Area of residence<br>- Health insurance
49	Koris et al	Socio-demographic, Cognitive Status and Comorbidity Determinants of Catastrophic Health Expenditure among Elderly in Malaysia	2017	Malaysia	Catastrophic spending	- Age<br>- Location<br>- Ethnicity<br>- Income<br>- Cancer prevalence
50	Mchenga et al	Impoverishing effects of catastrophic health expenditures in Malawi	2017	Malawi	Catastrophic spending	- Income<br>- Location
51	Miao et al	Multi-dimensional vulnerability analysis on catastrophic health expenditure among middle-aged and older adults with chronic diseases in China	2022	China	Catastrophic spending	- Poverty<br>- Malignant tumor<br>- IPD care<br>- Type of health insurance

52	Mohanty et al	Old-age dependency and catastrophic health expenditure: Evidence from Longitudinal Ageing Study in India	2022	India	Catastrophic spending	- Income, poverty<br>- Old-age dependency<br>- Ethnicity<br>- Location
53	Molla et al	Predictors of high out-of-pocket healthcare expenditure: an analysis using Bangladesh household income and expenditure survey,<br>2010	2017	Bangladesh	OOPE	- Chronic illness<br>- Household income<br>- Family size<br>- Presence of health shock
54	Sarker et al	Out-of-pocket payment for healthcare among urban citizens in Dhaka, Bangladesh	2022	Bangladesh	OOPE for acute and chronic illness	**OOPE for acute illness**<br>- Marital status<br>- Religion<br>- Source of care<br>- Access to safe water<br>- Income quintiles<br>**OOPE for chronic illness**<br>- Sex<br>- Religion<br>- Educational status<br>- Source of care<br>- Ownership of households<br>- Access to safe water<br>- Mass media access<br>- Regional differences
55	Sayuti et al	Assessing progress towards Sustainable Development Goal 3.8.2 and determinants of catastrophic health expenditures in Malaysia	2022	Malaysia	Catastrophic spending	- HoHH age <60<br>- Female HoHH<br>- Rural area<br>- Small HH size (1-2 members)
56	Yadav et al	Disease-Specific Out-of-Pocket Payments, Catastrophic Health Expenditure and Impoverishment Effects in India: An Analysis of National Health Survey Data	2021	India	Catastrophic spending	- Type of provider<br>- Type of disease (cancers, cardiovascular diseases, psychiatric conditions, injuries, musculoskeletal and genitourinary conditions)
57	Zhen et al	A comparative study of catastrophic health expenditure in Zhejiang and Qinghai province, China	2018	China	Catastrophic spending	<u>Reduced risk</u><br>- HoHH employed<br>- Status of HHs<br><u>Increased risk</u><br>- HoHH minor

						- Poor HH<br>- Low-insured HH<br>- Number of members with chronic diseases<br>- HH having outpatients<br>- Number of inpatients in HH
58	Haakenstad et al	Catastrophic health expenditure on private sector pharmaceuticals: a cross-sectional analysis from the state of Odisha, India	2022	India	OOPE per OPD visit<br><br>OOPE per IPD visit<br><br>OOPE budget share<br><br>Catastrophic spending<br><br>Distress financing	**Determinants of OOPE per OPD visit**<br>Increased OOPE per OPD visit<br>- Rural areas<br>- Chronic diagnosis<br>- Increased share with primary education or less in HH<br>- Days of school/work missed in past 15 days due to illness<br>- Illness: childbirth, other/none<br>- Number of medicines obtained for OPD visit<br>- OPD medicines obtained in private sector<br>Decreased OOPE per OPD visit<br>- Lower level of care (primary, other)<br><br>**Determinants of OOPE per IPD visit**<br>Increased OOPE per IPD visit<br>- Rural areas<br>- Wealth quintile<br>- Private IPD care<br>Decreased OOPE per IPD visit<br>- Scheduled tribe<br>- Use of insurance for hospitalization<br>- Illness: diarrhea, fever<br><br>**Determinants of OOPE budget share**<br>Increased OOPE budget share<br>- Rural areas<br>- Chronic diagnosis<br>- Increased share with primary education or less in HH<br>- Days of school/work missed in past 15 days due to illness<br>- Illness: childbirth, fever<br>- Number of OPD visits in last 15 days<br>- Number of hospitalizations in past 12 months<br>- Number of medicines obtained for OPD visit<br>- OPD medicines obtained in private sector

						Decreased OOPE budget share<br>- Increasing wealth<br>- Used insurance for hospitalization<br>- Lower level of care (primary, other)<br><br>**Determinants of catastrophic spending**<br>Increased catastrophic spending<br>- Rural areas<br>- Increased share with primary education or less in HH<br>- Days of school/work missed in past 15 days due to illness<br>- Illness: Childbirth<br>- Number of OPD visits in last 15 days<br>- Number of hospitalizations in past 12 months<br>- Number of medicines obtained for OPD visit<br>- OPD medicines obtained in private sector<br>Decreased catastrophic spending<br>- Wealth quintile<br><br>**Determinants of distress financing**<br>Increased distress financing<br>- Rural areas<br>- Days of school/work missed in past 15 days due to illness<br>- Illness: injury<br>- Number of OPD visits in last 15 days<br>- Number of hospitalizations in past 12 months<br>Decreased distress financing<br>- Wealth quintile<br>- Scheduled tribe
59	Bedado et al	Magnitude and determinants of out of pocket health expenditure among patients visiting outpatients in public hospitals in East Shoa Zone, Ethiopia	2022	Ethiopia	OOPE	Increased OOPE<br>- Respondents aged 31-49<br>- Secondary education and above<br>- Monthly income >4000 ETB<br>- Urban residents<br>- Family size
60	Onwujewke et al	Investigating determinants of out-of-pocket spending and strategies for coping with payments for healthcare in southeast Nigeria	2010	Nigeria	OOPE	Increased OOPE<br>- Age<br>- Family size<br>- Sex

						- Cost of treatment<br>- Transport costs
61	Mamani et al	Determinants of Out-of-Pocket Health Spending in Households in Peru in the Times of the Pandemic (COVID-19)	2023	Peru	OOPE	<u>Increased OOPE</u><br>- Type of insurance<br>- Results of COVID-19 tests<br>- Expenditure on individual health<br>- Existence of permanent limitations to any member of the household<br>- Presence of an older adult in the household<br>- Marital status of HoHH<br><br><u>Decreased OOPE</u><br>- Existence of drinking water service in household<br>- Educational level<br>- Area of residence
62	Du et al	Socioeconomic determinants of out-of-pocket pharmaceutical expenditure among middle-aged and elderly adults based on the China Health and Retirement Longitudinal Survey	2018	China	OOPE on pharmaceuticals (OPD, IPD, self-medication)	<u>Increased OOPE for OPD</u><br>- Above 65<br>- Female<br>- Type of insurance<br>- Self-reported health status<br>- ADL<br>- One or multiple chronic diseases<br>- Critical disease (severe)<br><br><u>Increased OOPE for IPD</u><br>- Increased education<br>- Type of insurance<br>- Income group<br>- Area of residence<br>- Self-reported health status<br>- Multiple chronic diseases<br>- Critical disease

Abbreviations: HH = household; HoHH = head of household; IPD = inpatient care; OOPE = out-of-pocket expenditure; OPD = outpatient care.
